# The Nature of Interface Interactions Leading to High Ionic Conductivity in LiBH_4_/SiO_2_ Nanocomposites

**DOI:** 10.1021/acsaem.2c00527

**Published:** 2022-06-16

**Authors:** Sander F. H. Lambregts, Ernst R. H. van Eck, Peter Ngene, Arno P. M. Kentgens

**Affiliations:** † Magnetic Resonance Research Center, Institute for Molecules and Materials, Radboud University, 6525 AJ Nijmegen, The Netherlands; ‡ Materials Chemistry and Catalysis, Debye Institute for Nanomaterials Science, Utrecht University, 3584 CG Utrecht, The Netherlands

**Keywords:** solid-state electrolyte, lithium borohydride, nanoconfinement, silica, solid-state NMR

## Abstract

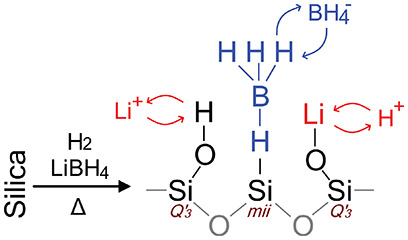

Complex metal hydride/oxide nanocomposites are a promising class of solid-state electrolytes. They exhibit high ionic conductivities due to an interaction of the metal hydride with the surface of the oxide. The exact nature of this interaction and composition of the hydride/oxide interface is not yet known. Using ^1^H, ^7^Li, ^11^B, and ^29^Si NMR spectroscopy and lithium borohydride confined in nanoporous silica as a model system, we now elucidate the chemistry and dynamics occurring at the interface between the scaffold and the complex metal hydride. We observed that the structure of the oxide scaffold has a significant effect on the ionic conductivity. A previously unknown silicon site was observed in the nanocomposites and correlated to the LiBH_4_ at the interface with silica. We provide a model for the origin of this silicon site which reveals that siloxane bonds are broken and highly dynamic silicon–hydride–borohydride and silicon–oxide–lithium bonds are formed at the interface between LiBH_4_ and silica. Additionally, we discovered a strong correlation between the thickness of the silica pore walls and the fraction of the LiBH_4_ that displays fast dynamics. Our findings provide insights on the role of the local scaffold structure and the chemistry of the interaction at the interface between complex metal hydrides and oxide hosts. These findings are relevant for other complex hydride/metal oxide systems where interface effects leads to a high ionic conductivity.

## 1 Introduction

All-solid-state batteries are expected to play an important role in the energy storage demands of our future society. Compared to traditional Li-ion batteries, which contain organic-liquid-based electrolytes, the electrolyte in an all-solid-state battery is based on polymers and/or inorganic salts. This mitigates the safety issues commonly associated with organic liquid-based electrolytes, such as evaporation of the flammable organic solvents.^1,2^ Furthermore, most all-solid-state batteries have an improved stability against dendrite formation, making them less susceptible to internal short circuiting. Due to their improved stability, solid-state electrolytes are often compatible with high-capacity electrodes, including metallic lithium, allowing for an improved energy density.

Among the potential all-solid-state battery electrolyte candidates is the class of complex metal hydrides.^3−5^ Complex metal hydrides form a stable interface layer against metallic lithium and display high ionic conductivities and negligible electronic conductivity. One particular complex metal hydride, lithium borohydride (LiBH_4_), has received significant attention since the discovery of fast ionic motion at elevated temperatures.^6^ Unfortunately, the ionic conductivity of bulk LiBH_4_ is poor below its structural phase transition, at 110 °C.

The room temperature ionic conductivity of complex metal hydrides has to be significantly improved for use as solid electrolytes in battery applications. Various authors have shown that partial ionic substitution of the anion or cation of LiBH_4_^7−9^ and the use of other (boro)hydride anions^5,10,11^ are promising routes to increase the room temperature ionic conductivity.

The other commonly used method to increase the room temperature ionic conduction of complex metal hydrides is to place the hydride in contact with an oxide surface, such as silica, magnesia, or alumina.^12−14^ The increased conductivity is known to be the result of an interface effect.^15−18^ The mobility of the ions near the oxide surface increases drastically compared to the bulk material. Nanoconfinement by infiltration in porous oxides as well as mechanochemical synthesis (ball-milling) are promising techniques to enhance the contact interface between the oxide and the complex metal hydride.^12,14,16^ Both techniques can be combined with other methods, such as ionic substitution, to even further enhance ionic conduction.^19,20^

Porous silicas (such as MCM-41 and SBA-15) typically have larger surface areas than silicas after mechanochemical treatment (ball-milling).^16,21^ This increases the available interface area of the oxide with complex metal hydrides. Furthermore, their long channels allow for continuous pathways of ionic conduction with little interparticle voids. However, these are both macroscopic parameters, with a macroscopic influence on ion conduction. Porous silica scaffolds have many other parameters, such as the pore diameter, surface roughness, and pore interconnectivity. The influence of these parameters on the interface interaction with confined complex metal hydrides has not been fully explored yet.

Recently, we have extensively studied the dynamics of LiBH_4_ in silica nanopores.^18^ In the present paper, we use solid-state NMR to study the interface between complex metal hydrides and oxides as well as the influence of the local oxide structure on the interaction of complex metal hydrides with this oxide scaffold. Lithium borohydride confined in nanoporous silica was used as a model system in order to elucidate how the oxide scaffold affects the dynamic behavior of a nanoconfined complex metal hydride. It is expected that the results presented in this paper can be generalized to other complex metal hydride/oxide systems, including nanocomposites prepared via mechanochemical synthesis.

## 2 Experimental Section

### 2.1 Sample Preparation

A short overview of properties of the nanocomposites described individually in the main text is listed in Table 1. Detailed synthesis protocols can be found in the Supporting Information. Fumed silica (Aerosil 90, 300 and 380) was commercially obtained.

**Table 1:** Properties of the Nanocomposites Described Individually in This Paper As Studied with NMR^a^

nanocomposite	area (m^2^/g)	pore volume (mL/g)	diameter (nm)	thickness (nm)	LiBH_4_ loading (%)	
MCM-41-C_16_-150^i^	1083	0.96	4.5	0.3	90	(37 wt %)
MCM-41-C_16_-150-2^i^	1194	1.06	4.5	0.4	90	(39 wt %)
MCM-41-C_16_-170^i^	474	0.39	5.1	0.9	91	(19 wt %)
MCM-41-C_16_-180^i^	346	0.33	4.6	1.6	93	(17 wt %)
MCM-41-C_16_-180-2^i^	421	0.39	4.7	1.6	91	(19 wt %)
SBA-15-85^i^	793	0.72	9.2	0.4	91	(30 wt %)
Aerosil 300^i^	278	ND	N/A	N/A	N/A	(29 wt %)

a More properties and the properties of the other nanocomposites studied by NMR can be found in the Supporting Information. The scaffold surface area, total meso- and micropore volume, mesopore diameter, pore wall thickness, and LiBH_4_ loading with respect to the pore volume are listed. The nomenclature of the samples is described in the text below.

SBA-15^22^ consists of long, parallel mesopores, with micropores and secondary mesopores in the pore walls.^23,24^ The primary mesopore diameter can be varied by tuning the hydrothermal synthesis temperature.^25^ The silicas are referred to as SBA-15 followed by their hydrothermal treatment temperature.

MCM-41^26^ consists of long, parallel mesopores, without secondary pores. The pore diameter was tuned by the choice of surfactant. Synthesis was performed as described by Cheng et al.^27^ Thick-walled MCM-41 was synthesized by using elevated hydrothermal treatment temperatures.^28^ The silicas are referred to as MCM-41 followed by the surfactant chain length (C_*n*_) and the hydrothermal treatment temperature; the suffix “-2” is used to differentiate scaffolds prepared using the same procedure.

Previously, we have shown that a drying pretreatment of the silica is crucial for ionic conduction in LiBH_4_/SiO_2_ nanocomposites.^29^ Consequently, all scaffolds were dried for 6 h at 300 °C under a flow of inert gas and subsequently stored in a glovebox.

The LiBH_4_/SiO_2_ nanocomposites were prepared via melt-infiltration of the dried silica scaffolds.^30^ Melt-infiltration under H_2_ has been shown to yield a good infiltration efficiency with little decomposition of the lithium borohydride. The LiBH_4_ loadings (fraction of pores that would be filled with LiBH_4_ if all LiBH_4_ infiltrates) can be found in Tables S2 and S3 for the samples used for impedance spectroscopy and NMR experiments, respectively. Nanocomposites used for studies by NMR are named by their scaffold suffixed by a superscript i or ii to distinguish nanocomposites of different batches that use the same silica scaffold.

### 2.2 Characterization of the Silica Scaffolds

The physical properties of the silica scaffold were characterized using N_2_-physisorption and powder X-ray diffraction (XRD). The properties of the silicas can be found in Table S1.

N_2_-physisorption isotherms of the dried silicas (Figure S1) were obtained at −196 °C on a Micromeritics TriStar surface area and porosity analyzer to determine the surface area, the pore size distribution (Figure S2), the total pore volume, and the micro- and mesopore volumes.

Low angle powder XRD measurements (Figure S3) were performed in air, using mica as the internal reference with its first large peak fixed at 10.45° 2θ. Diffractograms were recorded on a Bruker D2 Phaser diffractometer using Co Kα_12_ radiation (λ = 0.179026 nm). The distance between the centers of adjacent pores *a*_0_ was obtained from the (100) or (200) reflections in accordance with literature.^22^ The thickness of the silica pore walls *t* was obtained by subtracting the pore diameter (as obtained by physisorption) from *a*_0_.^22^

Diffuse reflectance infrared (DRIFTS) spectra were obtained on a PerkinElmer Frontier FT-IR spectrometer in a closed, argon-filled chamber at room temperature. Background spectra were subtracted from the spectra of the sample.

Transmission electron micrographs (Figure S4) were captured using a FEI Tecnai T20 transmission electron microscope operating at 200 kV to visually confirm the presence of mesopore ordering. Silica was suspended in isopropanol by sonification and then drop-casted on a holey carbon TEM grid prior to imaging.

### 2.3 Degree of Confinement

The degree of confinement of LiBH_4_ in the silica pores after melt-infiltration was determined using differential scanning calorimetry (DSC), where the degree of confinement is derived from the enthalpy of the structural phase transition of residual (extraporous) bulk LiBH_4_.^12^ LiBH_4_ fills pores completely, meaning pores are either empty or completely full.^31^ The procedure used follows that in our previous paper, where it was shown to be quantitative for SBA-15-based nanocomposites.^29^

### 2.4 Conductivity Measurements

Electrochemical impedance spectroscopy was performed using a Princeton Applied Research Parstat 2273 potentiostat in a Büchi B-585 glass oven under argon atmosphere using a custom-made measurement cell. Lithium foil (99.9%, 0.38 mm thick and 12 mm in diameter) was placed on top of two 13 mm stainless steel rods. The nanocomposite or LiBH_4_ was placed between the two stainless rods in a pellet die, such that it was in contact with the Li foil. After that, the sample was pressed using a pressure of 75 or 150 MPa. A 20 mV rms modulated AC potential with frequencies from 1 MHz to 1 Hz was applied to the compressed sample. The samples were heated at 5 °C/min to the desired temperature and allowed to equilibrate for at least 45 min before measurement.

A single, slightly depressed semicircle was observed in all cases in the Nyquist plots. In line with a previous report,^12^ the data were fitted using an equivalent circuit consisting of a resistance and a constant phase element. Consequently, the intersection of the fitted semicircle with the real impedance axis was assumed to represent the electrolyte resistance *R* only, and this value was related to the conductivity σ at that temperature via σ = *h*/*AR*, using the geometric area of the samples (*A* = 1.33 cm^2^) and the thickness *h* of the pellet (excluding Li foil).

### 2.5 Solid-State NMR Measurements

Solid-state NMR experiments were performed on 7.05 and 9.39 T Varian VNMRS spectrometers using Bruker 4.0 mm MAS, Bruker 5.0 mm static and Chemagnetics 7.5 mm MAS, and Varian 3.2 mm MAS T3 and RevolutionNMR 6.0 mm MAS probes, respectively. Experiments were performed under a flow of nitrogen due to the reactive nature of the samples. No changes have been observed in the samples over time. The ^29^Si single pulse spectrum of the dry silica scaffold MCM-41-C_16_-150-2 was measured in a rotor filled with dried air, although the MAS gas was N_2_.

Static ^1^H and ^7^Li spectra were recorded using radiofrequency (RF) field strengths of 60–80 kHz. ^1^H, ^7^Li, and ^29^Si MAS experiments and the ^11^B REDOR experiment utilized RF field strengths between 30 and 55 kHz for both excitation and cross-polarization (CP) contact pulses. The ^11^B single-pulse experiment used an RF field strength of 100 kHz. SPINAL ^1^H decoupling^32^ at an RF field strength of 55 kHz was applied in the static ^7^Li experiments, 80 kHz in the ^11^B single-pulse experiment, and 20–50 kHz in ^29^Si-detected MAS experiments. ^1^H decoupling was also applied during the echo time in echo experiments.

Single-pulse experiments (SPE) and solid echo experiments^33^ used 90° pulses, except ^11^B SPE experiments, where 30° pulses were used; Hahn echo experiments^34^ used a 90° and a 180° pulse. The delay between the excitation and echo pulse in echo experiments was 1 rotor period in MAS experiments and 14 μs otherwise. Saturation recovery experiments were used to determine spin–lattice relaxation times (T_1_). {^1^H}^29^Si cross-polarization (CP) experiments^35^ used contact times of 4 ms, with the exception of the variable time Lee–Goldburg cross-polarization (LGCP) experiments.^36,37^ The inverse detection {{^1^H}^29^Si}^1^H experiment^38^ used regular CP for the ^1^H-to-^29^Si transfer and LGCP for the reverse magnetization transfer. REDOR experiments^39^ used CP for the ^1^H-to-^29^Si transfer and alternating 180 ° pulses on ^29^Si and either ^11^B or ^7^Li during the recoupling time.

NMR spectra were processed and referenced according to the methods outlined in our previous paper.^18^ REDOR curves were fitted to the analytical formula derived by Hirschinger to obtain the heteronuclear dipolar second moment and the average internuclear distance.^40^

## 3 Results

### 3.1 Overall Lithium-Ion Conduction in Silica Scaffold Structures

**Figure 1:**
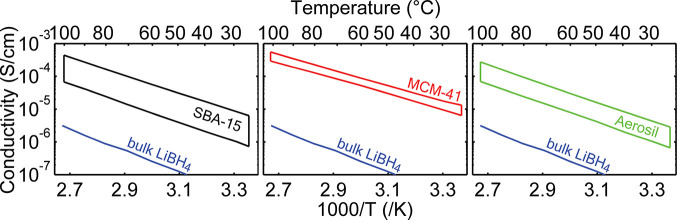
Arrhenius plots showing the ranges where the ionic conductivities of multiple different LiBH_4_/SiO_2_ nanocomposites are found, grouped by their silica scaffold structure, as a function of temperature. The red area surrounds all ionic conductivities of MCM-41-based nanocomposites, the black area corresponds to the SBA-15-based nanocomposites, and the green area corresponds to fumed silica (Aerosil) based nanocomposites. For clarity, only the outlines of the areas are shown. The composition and conductivity of the individual nanocomposites can be found in Table S2, and the individual Arrhenius plots can be found in Figure S5. The ionic conductivity of bulk LiBH_4_ is shown in blue.

Figure 1 shows Arrhenius plots of the ionic conductivities of LiBH_4_ confined in silica scaffolds. For clarity, the data for different samples are represented as areas, each covering all samples of the three different overall types of silica (SBA-15, MCM-41, and fumed silica). The ionic conductivity of bulk LiBH_4_ is also shown. The ionic conductivity of all of the nanocomposites exceeds that of bulk LiBH_4_ by over an order of magnitude, in agreement with literature.^12^ The ionic conductivity also depends on the structure of the silica used, with the general trend being that the nanocomposites utilizing MCM-41 silica perform on average better than the nanocomposites using SBA-15 or fumed silica scaffolds.

Conductivity measurements using only impedance spectroscopy cannot reveal the origin of these differences. This technique is sensitive to the net mobility of the Li^+^ ions throughout the sample, rather than individual (structural) contributions that each affect the ionic conduction, such as differences in surface area, particle dimensions and pore structure of the silica host. Furthermore, it requires an uninterrupted pathway between the electrodes for the lithium ions to diffuse through. This pathway for ion diffusion depends on the mixing of the materials prior to melt-infiltration and the compression of the pellet, e.g., the void space between the particles and interparticle contact. To study the influence of the silica at the LiBH_4_/SiO_2_ interface, the remainder of this paper focuses on studies using NMR as a characterization tool, which is sensitive to the local environment of the (NMR-active) isotopes and hence their mobility.

### 3.2 Lithium-Ion Mobility of Nanoconfined Lithium Borohydride

**Figure 2:**
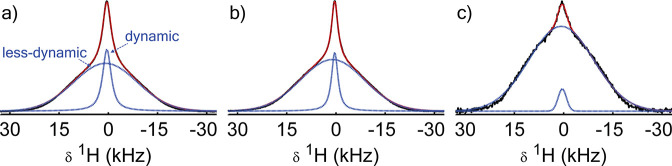
Static ^1^H NMR spectra of LiBH_4_/SiO_2_ nanocomposites (a) SBA-15-85^i^, (b) MCM-41-C_16_-150^i^, and (c) Aerosil 300^i^, measured at 30 °C in a field of 7.05 T using a single excitation pulse. The deconvolution in two individual peaks, corresponding to more-dynamic and less-dynamic ions, is shown with dashed blue lines, and the resulting fit is shown in red. The sample compositions can be found in Table S3.

Figure 2 shows the static ^1^H spectra of LiBH_4_/SiO_2_ nanocomposites at 30 °C. All spectra consist of a broad and a narrow component, indicative of two fractions of LiBH_4_ with different mobilities.^15^ In our previous paper, we have elaborated on the dynamics of LiBH_4_ within the silica pores.^18^ The fraction with the highest mobility, as reflected by the narrow peak, hereafter called the dynamic fraction, consists of Li^+^ and BH_4_^–^ ions that rapidly diffuse in an amorphous environment near the silica pore walls. Mechanisms like the so-called paddle wheel mechanism may further enhance the dynamics.^41,42^ The less-dynamic fraction, as reflected by its broad peak, resembles bulklike LiBH_4_ residing in the core of the silica pores, away from the silica pore walls. At temperatures below the structural phase transition of confined LiBH_4_ (as used throughout the present study), exchange between these fractions is slow for both Li^+^ and BH_4_^–^ ions.^18^

We showed that the ratio between the dynamic and less-dynamic ^1^H fractions is directly proportional to the distribution of Li^+^ over the dynamic or less-dynamic fractions.^18^ Hence, the ratio of the proton fractions is an excellent reflection of the overall distribution of the dynamic and less-dynamic fractions of nanoconfined LiBH_4_. It is experimentally convenient to utilize the proton line shapes for quantification of the fraction of highly dynamic ions, as proton NMR is more sensitive than lithium-6 NMR, and its line shapes are not complicated by the presence of quadrupolar interactions as is the case for lithium-7.

Table S3 lists the fraction of highly dynamic LiBH_4_ at 30 °C for each of the nanocomposites analyzed by ^1^H NMR. The corresponding ^1^H and, for reference, ^7^Li spectra are shown in Figures S6 and S7. The individual nanocomposites display mobile fractions of 13–53% of the LiBH_4_ at 30 °C. These numbers correspond well with previously reported studies of LiBH_4_/SiO_2_ nanocomposites.^12,15,18^ In contrast, only 3–4% of the LiBH_4_ is highly dynamic in the nanocomposites of LiBH_4_ and fumed silica.

Nanoconfinement in mesoporous silica thus generally leads to higher fractions of mobile LiBH_4_ compared to ball-milled LiBH_4_/SiO_2_ nanocomposites. This leads to the question: what properties of the silica scaffold influence the fraction of highly dynamic confined LiBH_4_? Previous research showed that the drying pretreatment of the silica (and thus the surface groups of the silica) has a major effect on the conductivity.^29^ To eliminate this effect, all silica scaffolds in this study were given an identical drying pretreatment prior to infiltration.

Geometrically, the scaffold surface area (which can reach over 1000 m^2^/g in mesoporous silica scaffolds^26^) is expected to strongly influence the total amount of LiBH_4_ in contact with the surface. During the melt-infiltration process, the nanopores of the silica scaffolds are completely filled with LiBH_4_. As only lithium borohydride near the interface with silica is highly dynamic,^17,18,43^ the fraction of highly dynamic LiBH_4_ is expected to decrease when the pore size increases. Previous research indeed suggests an influence of the pore diameter on the fraction of highly dynamic LiBH_4_: when varied between 5 and 8 nm, a difference in the relative amount of highly dynamic LiBH_4_ of about 10% was observed, in favor of the more narrow pores.^18^

The aforementioned surface area of the silica scaffolds is a macroscopic property, and therefore, its influence is not expected to be directly visible in NMR spectra. However, also the mesopore diameter can insufficiently explain the differences in the observed ratio of dynamic and less-dynamic ions among nanocomposites with varying silica scaffolds (Figure S8). A similar observation was made by de Kort et al., who found no clear correlation between the (macroscopic) ionic conductivity and the pore diameter of the scaffold.^20^ Varying only the pore diameter without influencing other properties of the scaffold is difficult. For example, the amount of secondary (micro)pores in SBA-15 strongly correlates with the synthesis conditions, and varying the mesopore diameter will therefore also influence the pore corrugation.^23−25^ Hence, we quantitatively restrict this study to a selected series of MCM-41-based nanocomposites and only discuss the other nanocomposites qualitatively.

### 3.3 Effect of LiBH_4_ Infiltration on the Silica Scaffold

The infrared signal of non-hydrogen-bonded (“isolated”) silanol groups on the surface of silica was found to disappear after melt-infiltration.^29^ In that study, it was not possible to determine whether these silanol groups could no longer be observed due to a change of interactions (such as a larger spread in the O–H bond length) or due to a chemical interaction (i.e., reaction or hydrogen bond formation). To overcome this limitation, we use ^29^Si CP-MAS NMR to probe interactions at and changes of the silica surface.

**Figure 3:**
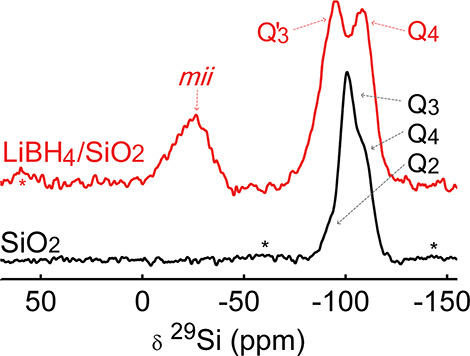
Normalized ^29^Si CP-MAS NMR spectra of LiBH_4_/SiO_2_ nanocomposite MCM-41-C_16_-150-2^i^ and the corresponding silica (MCM-41-C_16_-150-2), showing the silanol and siloxane peaks (labeled Q_*n*_, see text) and the melt-infiltration-induced (*mii*) peak in the nanoconfined sample. The positions of the peaks in the top and bottom spectrum are representative for the nanoporous silicas with and without LiBH_4_, respectively. A contact time of 4 ms was used at a MAS speed of 3.25 (silica) or 6.5 kHz (nanocomposite) in a field of 9.4 T. Spinning sidebands are marked with asterisks.

The bottom spectrum in Figure 3 shows a typical ^29^Si NMR spectrum of silica scaffolds (without LiBH_4_). It displays three partly overlapping peaks at approximately −109, −101, and −91 ppm. These peaks correspond to the silicon in *Si*(O–Si−)_*n*_(OH)_4–*n*_ with *n* = 4, 3, or 2, respectively; hereafter denoted as Q_*n*_.^44,45^

The spectrum of the silica infiltrated with LiBH_4_ shows an additional, broad peak around −25 ppm. To our knowledge, such a peak has not been reported in the literature. Some organosilicates (e.g., D_*n*_-groups) can resonate in this spectral region,^46^ yet their absence in the ^29^Si NMR spectra of the parent silicas suggests these species are not present. This is corroborated by the absence of C–H vibrations in infrared spectra of the silica scaffolds (Figure S9). We will refer to this new peak in the ^29^Si NMR spectrum as the melt-infiltration-induced (*mii*) peak.

Besides the additional peak, also the region where the Q_*n*_ peaks resonate has changed upon melt-infiltration. The chemical shift of the Q_3_ peak increases to −95 ppm. This increase could be indicative of an increase in the Si–O bond length of these silanol groups^47^ or a change at the site of the proton. The Q_3_ peak of the nanocomposites will hereafter be referred to as Q′_3_ to distinguish it from the Q_3_ peak in native silica.

CP-MAS NMR spectra are not inherently quantitative, as the signal intensity in these experiments depends on the proximity of protons to the observed nucleus. In case of silica, this leads to an overestimation of the silanol groups (which contain protons by definition) and silicon atoms in the proximity of these silanol groups. In the nanocomposites, the proximity of (proton-abundant) borohydride ions may additionally lead to an artificial increase of signals from the surface groups of the silica.

To overcome this limitation, single pulse excitation (SPE) experiments were used to quantify the silicon sites (Table 2 and Figure S10). From the spectrum of the dried silica scaffold, the silanol (Q_2_+Q′_3_):siloxane (Q_4_) ratio can be estimated as roughly 1:2. In the spectrum of the nanocomposite, this ratio is 1:1. Additionally, the *mii* peak corresponds to 16 ± 3% of the signal in the ^29^Si spectrum of this nanocomposite, implying that *mii* sites constitute a significant fraction of the silicon sites, rather than being a minority component with a high CP efficiency. The change in the ratios of the Q_n_ peaks shows that the Q_4_ (siloxane) groups are involved in the formation of the *mii* peak. Additionally, as only 16% of the intensity in the ^29^Si spectrum of the nanocomposite corresponds to *mii* sites, the change in the silanol:siloxane ratio (1:2 to 1:1) suggests that this conversion involves the formation of Q′_3_ sites too, i.e. some Q_4_ sites convert to Q′_3_ sites.

**Table 2:** Relative Abundance of Silicon Sites in a Dried Silica Scaffold (MCM-41-C_16_-150-2) before and after Infiltration with LiBH_4_^a^

	*mii*	silanol + Q′_3_	siloxane
silica		27 ± 9%	73 ± 9%
nanocomposite	16 ± 3%	41 ± 6%	42 ± 9%

a The NMR spectra can be found in Figure S10.

Both the *mii* peak around −25 ppm and the Q′_3_ peak are not observed in silica treated under melt infiltration conditions in the absence of LiBH_4_ (Figure S11). Hence, we can conclude that these spectral changes are due to the interaction between the silica and the LiBH_4_.

### 3.4 Interaction of LiBH_4_ with Silica

**Figure 4:**
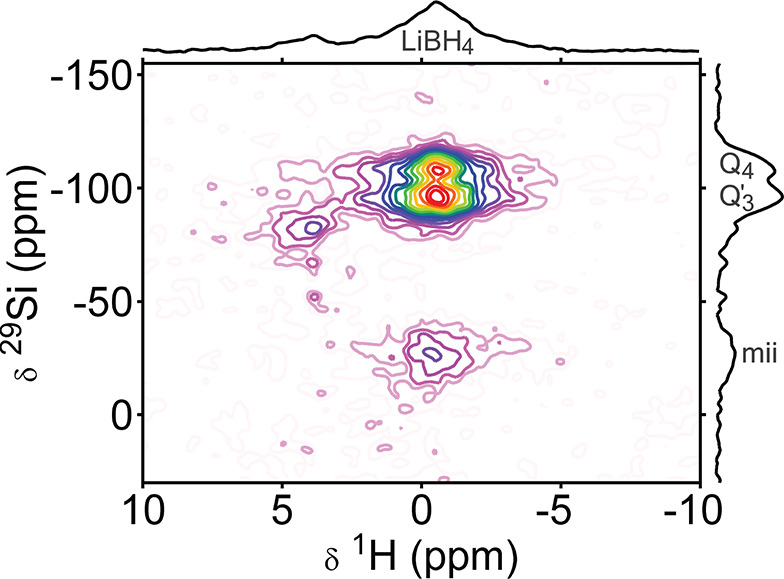
Two-dimensional {{^1^H}^29^Si}^1^H inverse detection heteronuclear correlation spectrum of a thin-walled MCM-41-based LiBH_4_/SiO_2_ nanocomposite (MCM-41-C_16_-150-2^i^), showing cross-peaks, indicative of close proximities between proton and silicon sites. The spectrum was recorded in a field of 9.4 T under 6.5 kHz MAS, using LGCP polarization transfer. Skyline projections are shown on the sides.

The interaction that manifests itself in ^29^Si NMR spectra of infiltrated silica scaffolds was further probed to determine the relation between the silicon sites and LiBH_4_. The two-dimensional correlation experiment between ^29^Si and ^1^H reveals which protons are in the immediate proximity of (specific) silicon atoms (Figure 4). The spectrum, shown in Figure 4, correlates the chemical shift of protons with those of the nearby silicon atoms. The skyline projection on the right of the Figure shows good similarity with the ^29^Si CP spectrum in Figure 3. The skyline projection at the top shows two ^1^H peaks. The right-most of those peak has the same proton chemical shift as LiBH_4_, at about −1 ppm, and is thus assigned to the protons in BH_4_^–^ groups.

The site with a ^1^H chemical shift of 4 ppm only correlates with ^29^Si sites at −83 ppm. As no peak is observed at 4 ppm in the ^1^H MAS spectrum of the silica scaffold before infiltration (Figure S12), it is unlikely that this peak corresponds to silanol groups or physisorbed water in the silica framework. Instead, we assign this combination of chemical shifts to the resonances of silicon hydride (Si–H), which will only be present after infiltration with LiBH_4_ (vide infra), and resonates at exactly these chemical shifts.^48^

Interestingly, the proton peak at −1 ppm (corresponding to BH_4_^–^) shows cross-peaks to all peaks that are also distinguishable in the ^29^Si CP-MAS spectrum. This proves that borohydride ions are in the vicinity of Q_4_, Q′_3_, and *mii* sites. In the correlation spectrum, the Q′_3_ peak couples only to the BH_4_^–^ peak. Silanol groups (including Q_3_) are expected to resonate between δ(^1^H) = 1 and 8 ppm.^49,50^ Very little spectral intensity in this range correlates with the ^29^Si chemical shift of Q_3_ or Q′_3_ groups. This additionally confirms that the majority of the Q_3_ groups have changed in the nanocomposite.

**Figure 5:**
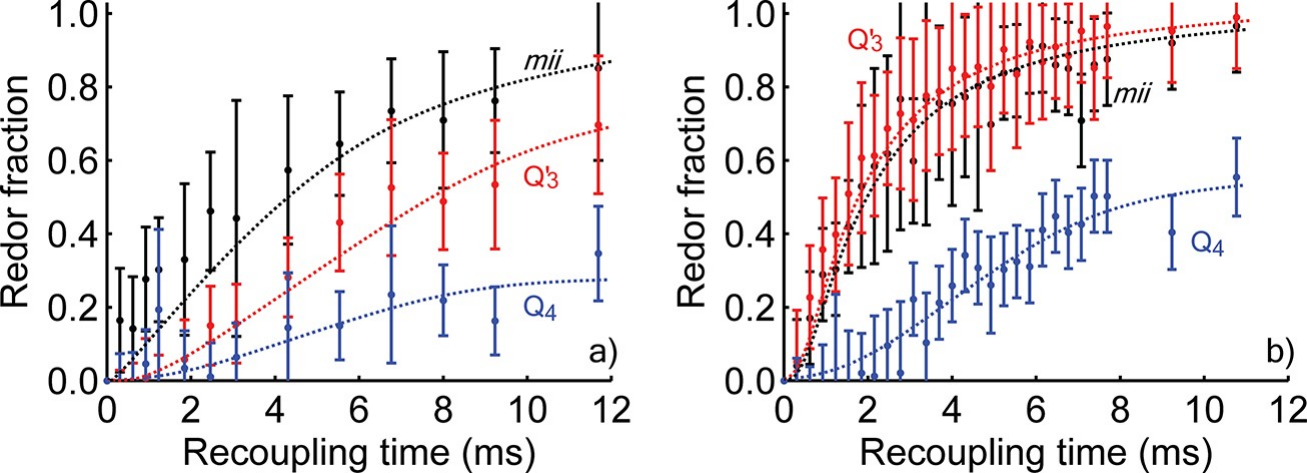
(a) {^11^B}^29^Si and (b) {^7^Li}^29^Si REDOR difference curves of the LiBH_4_/SiO_2_ nanocomposite MCM-41-C_16_-150-2^i^, measured at 9.4 T under 6.5 kHz MAS. The curve of Q′_3_ may include a contribution of isolated SiH and/or SiOH species, but this contribution is expected to be small. Lines were added to guide the eye only; fits can be found in the Supporting Information. For interpretation of REDOR curves, the reader is referred to the text.

The two-dimensional heteronuclear correlation spectrum reveals only whether a dipolar interaction exists between two sites. REDOR experiments are able to obtain the average distance between nuclei. Figure 5 shows the REDOR difference curves of boron and lithium with silicon. The buildup of these curves reflects the strength of the dipolar coupling between silicon and either boron or lithium, and therefore the distance between the nuclei.

In both the {^11^B}^29^Si and {^7^Li}^29^Si REDOR experiments (Figures 5a and b, respectively), the REDOR curves of the *mii* and silanol peaks approach unity at long recoupling times. Unity will be reached when all observed (^29^Si) spins are involved in a dipolar interaction with the nucleus that is recoupled. Hence, the vast majority of the *mii* silicon species must be coupled to both BH_4_^–^ and Li^+^.

Fits of the REDOR curves provide the *r*^–3^-weighted average distances between lithium or boron to silicon sites (Table S4). The average distance of the *mii* silicon site to either Li^+^ or BH_4_^–^ is very similar to the distance between the Q′_3_ site and Li^+^ (3.4 Å). In contrast, the average distances of BH_4_^–^ to the silicon of silanol or siloxane sites, or Li^+^ to siloxane sites, all exceed 4.5 Å. The latter distance is of the same order as the thickness of the shell of highly dynamic LiBH_4_ at ambient temperatures. Due to the *r*^–3^-dependency of the dipolar interaction, the contribution of silicon sites deeper in the silica framework can be neglected. It is safe to conclude that the observed Q_4_ (siloxane) groups are not directly involved in the interaction between silica and LiBH_4_, and Li^+^ is closer to the Q′_3_ site than BH_4_^–^ is. Also, all obtained distances significantly exceed the H···Si distance within a silanol group (roughly 2.3 Å), making a (nonexchanging) covalent bond between the silica scaffold and lithium or boron(hydride) unlikely.

As discussed, if the interaction between LiBH_4_ and the silica scaffold only depends on the surface area within the pore, it does not sufficiently describe the differences in the ratio of dynamic and less-dynamic LiBH_4_ in the pores of the different silica scaffolds (Figure S8). One of the things that has not been investigated is the influence of the pore wall thickness of the silica scaffolds, which differs also for SBA-15 and MCM-41 type scaffolds. Therefore, we also studied the effect of the thickness of the silica wall surrounding the pores.

### 3.5 Effects of Silica Pore Wall Thickness on LiBH_4_ Fractions

**Figure 6:**
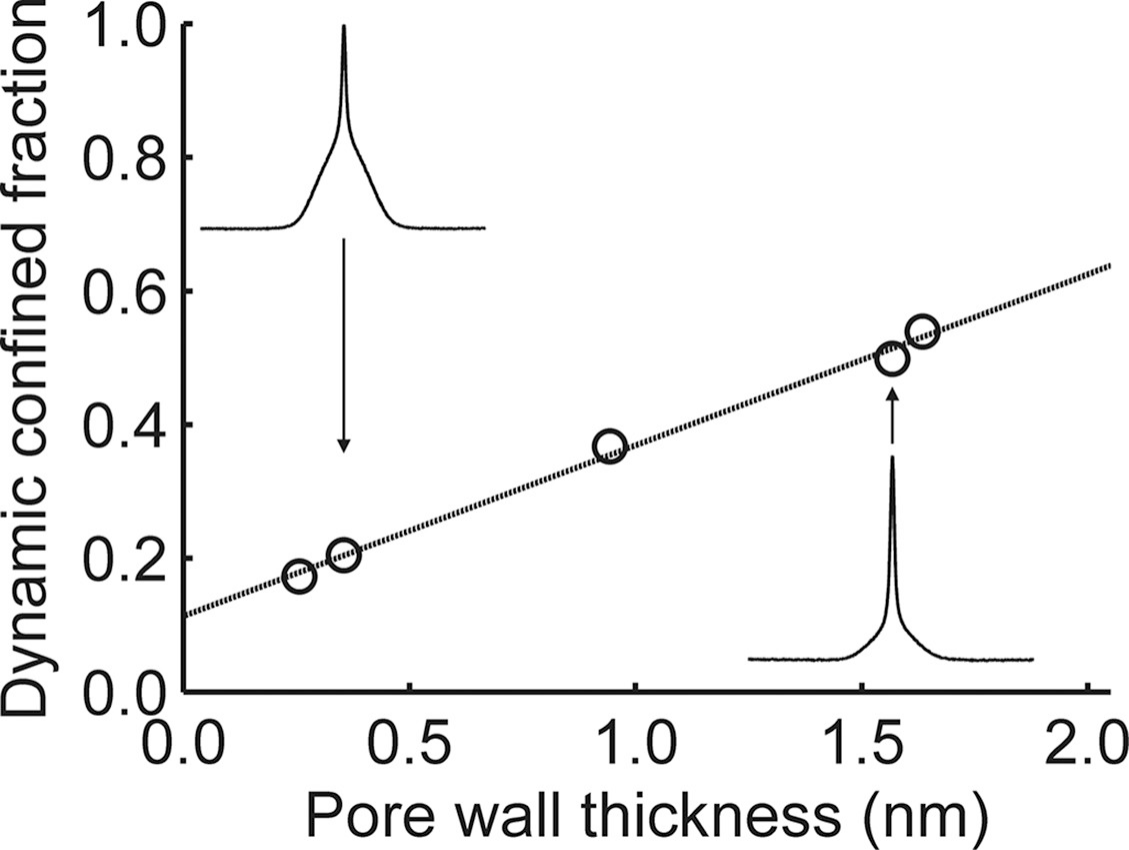
Fraction of LiBH_4_ that is highly dynamic on the NMR time scale, out of the total amount of LiBH_4_ that has been confined (as defined by DSC), plotted versus the pore wall thickness. The data points correspond to the fraction of protons in the highly dynamic fraction in MCM-41 type silica with (DFT) pore diameters between 4.5 and 5.1 nm. The dashed line is a least-squares fit. The pore wall thickness was derived using the DFT pore size and the (100) reflection in XRD. The apparent offset at a pore wall thickness of 0 (i.e., no silica walls) is most likely the result of the model used, and therefore without physical meaning, as demonstrated in Figure S14. The insets show the corresponding ^1^H spectra of nanocomposites MCM-41-C_16_-150-2^i^ and MCM-41-C_16_-180-2^i^.

Figure 6 shows the fraction of highly dynamic, confined LiBH_4_ as a function of the pore wall thickness of silica scaffolds. The points in this figure all correspond to LiBH_4_ nanoconfined in MCM-41-type silica scaffolds with nearly identical pore diameters, but with varying pore wall thicknesses: the thickness of the silica layer between two adjacent pores.

Surprisingly, the figure shows a clear trend where more lithium borohydride resides in the highly dynamic fraction when the pore wall thickness increases. At a pore wall thickness of about 0.4 nm, only 20% of the nanoconfined LiBH_4_ displays increased dynamics, whereas at a pore wall thickness of about 1.5 nm, the fraction of highly dynamic LiBH_4_ is almost three times larger.

The SiO_2_:LiBH_4_ ratio is commonly reported to have a strong influence on the ionic conductivity of LiBH_4_/SiO_2_ nanocomposites.^13,16^ However, it is important to note that this is a different phenomenon than what is probed by NMR. Ionic conductivity measurements using i.e. impedance spectroscopy rely on a continuous pathway of highly mobile ions. An excess of silica would lead to (nonconducting) voids in this conduction pathway, whereas an excess of LiBH_4_ would display the behavior of (poorly conducting) bulk lithium borohydride. NMR spectroscopy, on the other hand, detects the intrinsic, local dynamics of LiBH_4_. Hence, unlike impedance spectroscopy, it does not rely on a continuous pathway of conductive ions. As a consequence, the dependence of the fraction of mobile LiBH_4_ on the pore wall thickness (and thus, given that the silica structures are otherwise similar, the SiO_2_:LiBH_4_ ratio) as observed by NMR was not predicted. Hence, the trend shown in Figure 6 must have a different physical origin (vide infra).

{^1^H}^29^Si LGCP build-up curves of thick- and thin-walled MCM-41-based nanocomposites (Figure S15) reflect the rate of polarization transfer between proton and silicon nuclei: a more efficient (faster) transfer indicates a stronger dipolar coupling between the nuclei. In both thick and thin walled silica, the fastest buildup occurs for the *mii* peak, followed by the Q′_3_ peak, and finally the Q_4_ peak. These build-up curves are, within error, identical for the two nanocomposites. Hence, it is unlikely that the strength of the interaction between protons (either borohydride or silanol) and silicon nuclei causes the observed difference in the ratio of highly dynamic versus less dynamic LiBH_4_.

## 4 Discussion

Our results show that the fraction of highly dynamic LiBH_4_ in nanoporous silica is highly dependent on the pore wall thickness of the silica scaffold (and, consequently, the lithium borohydride-to-silica ratio). As NMR spectroscopy only probes the local dynamics of the ions, not relying on uninterrupted conduction pathways, such a dependency was unexpected.

The spectra also reveal a distinct chemical interaction between silica and lithium borohydride. This interaction manifests itself as a broad melt-infiltration induced (*mii*) resonance at δ(^29^Si) ≈ –25 ppm. The large change in chemical shift of this peak with respect to the Q_n_ peaks indicates that the environment of those silicon atoms has drastically changed. A rough calculation (outlined in the Supporting Information) suggests this peak is proportional to the amount of silica, rather than the surface area of the silica. Comparison of the Q′_3_:Q_4_ ratio in nanocomposites with the Q_3_:Q_4_ ratio in the silica suggests that the *mii* peak originates from a reaction involving Q_4_ sites.

It is unlikely that an irreversible reaction occurs between intact, highly mobile ions and the silica surface, due to the fast dynamics of both the lithium and the borohydride ions in the interface layer with the silica. Instead, rapidly (ex)changing coordination of Li^+^ or BH_4_^–^ ions with the surface of the silica scaffold is more likely. Coordination of lithium ions to an oxide scaffold was also proposed by Van Wüllen et al. in their study of lithium trifluoromethanesulfonate in alumina.^51^ Similarly, Breuer et al. proposed coordination of F^–^ ions in nanocomposites of lithium fluoride in alumina.^52^

A question of particular interest is what the nature of the *mii* site is. The chemical shift has increased (i.e., became less-negative) compared to the Q_*n*_ sites. It is even shifted further down than expected for silicon sites with one neutral alkyl group (T_*n*_ sites) instead of an electronegative oxygen-bound site.^46^ This suggests an interaction of silicon with a neutral or electropositive species.

A key discovery was the small quantity of isolated silicon hydride species in the infiltrated silica scaffold. Silicon hydride on silica is typically formed by reduction in H_2_ gas at temperatures far above those utilized during the melt infiltration procedure (800 °C, atmospheric pressure).^48^ However, the drying pretreatment temperature (300 °C) is known to lead to weakened Si–O–Si bonds on the surface of silica.^53^ The barrier to form Si–H sites is much lower at defect sites.^54^ Additionally, besides the approximately 100 bar H_2_ at 300 °C during infiltration, also (molten) LiBH_4_ is a strong reducing agent, capable of reducing Si–O–Si bonds to silicon hydrides.^55^

**Figure 7:**
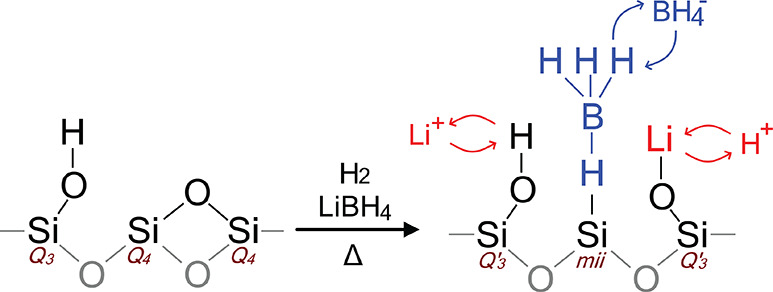
Two-dimensional model of the proposed structure near a *mii* site. A siloxane bond is reduced during infiltration. A BH_4_^–^ ion binds to a former Q_4_ site, forming a *mii* site. Li^+^ is exchanging with a silanol (Q_3_) site next to a *mii* site, leading to a Q′_3_ group. The model is not to scale but merely illustrates which sites interact. Curved arrows represent the mobility of the ions.

Hence, we propose that, during infiltration, some of the siloxane (Si–O–Si) bonds break in a reaction with the reducing agents. Herein, the Si^•^ would form a complex with a hydride and Si–O^•^ would form a Q′_3_ group. The BH_4_^–^ ion forms a complex with the hydride site, leading to Si···H···BH_3_ complexes. At the Q′_3_ site, exchange between the proton (Si–O–H, δ(^29^Si) ≈ −101 ppm) and lithium (Si–O–Li, δ(^29^Si) ≈ −87 ppm^56^) leads to a single Q′_3_ peak at the average chemical shift. This situation is schematically depicted in Figure 7. The proposed scheme (2Q_4_ → Q′_3_ + *mii*) corresponds very well with the changes in relative intensity of the silica sites before and after infiltration (Table 2).

Various authors have reported the release of hydrogen gas from nanocomposites prepared via wet infiltration and ball milling, assigned to the reaction between LiBH_4_ and the hydroxyl groups of the oxide scaffold, and proposed reactions forming Si–O–BH_3_.^57,58^ If hydrogen gas is formed, it is expected that the Q′_3_ groups in Figure 7 consist mostly of SiOLi. However, the nanocomposites in this study were prepared under H_2_ pressure, thereby shifting the equilibrium of reactions away from the formation of hydrogen gas.^30^ Nevertheless, the ratio and the extent to which the SiOH:SiOLi ratio affects the fraction of mobile ions have yet to be investigated.

Choi et al. proposed that boron-oxide species are responsible for the high ionic conductivity of LiBH_4_ nanoconfined in oxides.^59^ Although oxidized boron species are indeed observed in both ball-milled^59^ and melt-infiltrated^18^ nanocomposites (see also Figure S16), they represent only a small fraction (5–23% in total) of the ^11^B sites observed in the spectra of the various samples. Among the oxidized species are also boron oxides, such as B_2_O_3_. No clear correlation was discovered between the relative abundance of an oxidized species and the fraction of highly dynamic ions of LiBH_4_. Although some oxidation can be beneficial for the ionic conductivity of (nonconfined) complex metal hydrides,^60^ we expect this effect to be much less significant in nanoconfined LiBH_4_ due to the abundant silicon oxide and the absence of oxygen gas during synthesis. It is possible that boron and the oxygen of the Q′_3_ site interact, yet our REDOR experiments show that the coupling between boron and Q′_3_ is much weaker than the interaction of boron with the *mii* site. Furthermore, B–O bonds have also been observed in LiBH_4_/carbon nanocomposites.^61^ Consequently, we believe that the observed boron oxide species are not a vital component at the lithium borohydride/silica interface for the layer of highly dynamic LiBH_4_.

An alternative model would be lithiation (lithium infiltration) of silica. This process is commonly observed in silicon/silica-based anodes.^62−64^ Schnabel et al. have recently shown that the lithiation behavior of lithium in silica is strongly dependent on the thickness of the silica.^65^ Lithiation occurred readily in thin layers of silica, <2 nm in their system, whereas at thicknesses > 3 nm it was only observed around pinholes. Additionally, Freytag et al. have observed a peak centered around −29 ppm that they ascribe to a amorphous lithium silicide in a fully lithiated silicon mono-oxide sample.^66^ However, in LiBH_4_/SiO_2_ nanocomposites, lithiation as observed in these anodes is unlikely. Lithiation is known to also yield (electronically conducting) silicon, lithium silicates (Li_*x*_Si_*y*_O_*z*_), lithium silicides (Li_*x*_Si_*y*_), and lithium oxide, none of which has been identified in our ^29^Si or ^7^Li NMR spectra of LiBH_4_/SiO_2_ nanocomposites. Additionally, we have shown that the *mii* silicon site interacts with both Li^+^ and BH_4_^–^, but true infiltration of both ions through the silica is improbable given their opposite charges.

Surface interactions alone cannot explain the dependence of the fraction of LiBH_4_ that is highly dynamic on the thickness of the silica pore walls. This is corroborated by the LGCP experiment, which shows that the thickness of the pore walls has little effect on the strength of the interaction between silica and LiBH_4_ at room temperature.

In the absence of infiltration of LiBH_4_ into the silica, the dependence on the pore wall thickness can only have a charge-related origin. We postulate that thicker-walled silica is able to adsorb more charge. This may affect the stability of the transition state during the formation of the *mii* and Q′_3_ surface sites. Additionally, this would allow more charged ions to interact with the silica scaffold simultaneously. Indeed, Sen and Barisik calculated that a thicker pore wall (corresponding to a lower internal porosity in their paper) has a stronger electric double layer (EDL) effect in the core of pores due to overlap of the double layers of the opposing pore walls.^67^

Similar EDL overlap effects are present at the sites of connecting silica pores^68^ and rough surfaces^69^ (micropores). These variations in the EDL may explain why nanocomposites utilizing SBA-15 and MCM-41 yield different conductivities. A systematic study of this effect would be required to confirm this correlation. Considering the effects of the pore structure on the electric potential near the silica pore wall shows that the interface effect between LiBH_4_ and silica is clearly not simply a matter of a large surface area. Instead, the amount of highly dynamic ions depends on the exact structure of the porous material.

An increase in pore wall thickness will increase the amount of mobile ions in the pores of the nanoscaffold. It should however be noted that, for use in a battery, an uninterrupted conductive pathway is still required. Thicker pore walls will also make it less convenient to compress the silica particles into a stable, void-free pellet (exemplified in Figure S17). Hence, direct comparison of ionic conductivities of different samples should be done with great care. We believe that these results also apply to ball-milled nanocomposites, where the optimal oxide (such as silica) to complex metal hydride (e.g., LiBH_4_) ratio and conductivity will depend not only on the relative amounts of the materials and the material treatment pre- and postmilling but also on the shape of the oxide particles after the mechanical treatment.

## 5 Conclusions

We have studied the dynamics and interactions at the interface of confined complex metal hydrides and oxides to unravel the effects of the local pore structure and the surface chemistry of the oxide on the ionic mobility of the hydride. Nanocomposites of lithium borohydride in silica (MCM-41, SBA-15, and fumed silica) serve as a model system. The results reveal that the structure of the silica scaffold has a strong effect on the ionic conductivity of LiBH_4_/SiO_2_ nanocomposites: those containing MCM-41-type silica reach higher ionic conductivities at room temperature than SBA-15 and fumed silica based nanocomposites.

Our study revealed a strong correlation between the thickness of the silica pore walls and the fraction of LiBH_4_ in the nanopores displaying fast dynamics. An increase in the pore wall thickness leads to a proportional increase of the fraction of highly dynamic LiBH_4_. This effect is expected to originate from charge distributions within the silica. To our knowledge, such a correlation has never been observed before.

^29^Si spectra of the nanocomposites reveal a new silicon site resonating at δ(^29^Si) ≈ −25 ppm. This silicon site is abundantly present in the interface region between silica and LiBH_4_ and is expected to be an important contributor to the enhancement of the ionic conduction of LiBH_4_ in silica. Using one- and two-dimensional ^1^H, ^7^Li, ^11^B, and ^29^Si solid-state NMR experiments, we determined the chemistry at the LiBH_4_/SiO_2_ interface and developed a model for the structure of this new silicon site. In this model, a siloxane (Si–O–Si) bond is reduced during the synthesis of the nanocomposite. Subsequently, BH_4_^–^ forms Si···H···BH_3_ complexes with the resulting silicon hydride bond. Simultaneously, Li^+^-ions exchange with protons at the silanol sites.

The results in this article provide a new view on the role of the scaffold structure and chemistry in LiBH_4_/SiO_2_ nanocomposites and complex metal hydrides in oxide scaffolds in general. We expect that further improvements of the ionic mobility in complex metal hydride/oxide nanocomposites can be achieved by increasing the silicon hydride concentration. Additionally, we have shown that the optimal ratio between complex metal hydrides and oxides for batteries depends strongly on the local structure of the oxide scaffold. These results should guide the development of complex metal hydrides electrolytes to yield a higher ionic conductivity and, thereby, a more effective all-solid-state battery.

## Supporting Information Available

Synthesis protocols of mesoporous silica scaffolds and nanocomposites; characterization of the silica scaffolds and nanocomposites (X-ray diffraction, physisorption, electron microscopy, impedance spectroscopy, calorimetry, infrared spectroscopy); additional ^1^H, ^7^Li, ^11^B and ^29^Si solid-state NMR spectra; fit results of REDOR curves; LGCP buildup curves. Graphs of pore diameter or wall thickness versus dynamic LiBH_4_; compressibility of the silica. Quantification of *mii* sites with respect to pore wall thickness (PDF)
