# Supplementary material for: The Nature of Interface Interactions Leading to High Ionic Conductivity in LiBH4/SiO2 Nanocomposites
Source: ACS Appl Energy Mater. 2022 Jun 16;5(7):8057–66. doi: 10.1021/acsaem.2c00527 (PMC9345629; doi:10.1021/acsaem.2c00527)
Supplement: Supplementary file 1 — ae2c00527_si_001.pdf [file ae2c00527_si_001.pdf]

Supporting Information: On the Nature of Interface Interactions Leading to High Ionic Conductivity in $LiBH_4$/ SiO_2 Nanocomposites

Sander F. H. Lambregts,[†] Ernst R. H. van Eck,[†] Peter Ngene,[‡] and Arno P. M. Kentgens[*,†]

†*Magnetic Resonance Research Center, Institute for Molecules and Materials, Radboud University, 6525 AJ, Nijmegen, The Netherlands.*

‡*Materials Chemistry and Catalysis, Debye Institute for Nanomaterials Science, Utrecht University, 3584 CG, Utrecht, The Netherlands.*

E-mail: a.kentgens@nmr.ru.nl

1 Synthesis protocols

1.1 Mesoporous silica scaffolds

SBA-15 The primary mesopore diameter of Santa Barbara Amorphous-15[S1] (SBA-15) can be varied by tuning the hydrothermal synthesis temperature.[S2] A mixture of poly(ethylene glycol)-block-poly(propylene glycol)-block-poly(ethylene glycol) (P123), hydrochloric acid (37 %) and water was dissolved at 35 °C. Tetraethyl orthosilicate (TEOS) was added dropwise and the mixture was stirred at 40 °C for 24 h. The molar ratios were 0.015:5.2:147:1 for P123:HCl:H_2O:TEOS respectively. Hydrothermal treatment was done by heating the mixture to 40, 70, 85, 100 or 120 °C in a closed bottle or autoclave for 44 h, after which the solid precipitate was washed with water. The product was dried, ground, and subsequently calcined in air by increasing the temperature to 550 °C with a ramp of 1.2 °C/min and kept at this temperature for 12 h. The silicas are referred to as SBA-15 followed by their hydrothermal treatment temperature.

MCM-41 The pore diameter of Mobil Composition of Matter 41[S3] (MCM-41) was tuned by the choice of surfactant. Synthesis was performed as described by Cheng et al.[S4] Thick-walled MCM-41 was synthesized by using elevated hydrothermal treatment temperatures (170-180 °C).[S5] A mixture of water, n-alkyl-trimethylammonium bromide (C_nTAB, n=10,14,16) and tetramethylammonium hydroxide (TMAOH, 25 %) was dissolved at 30 °C. Aerosil was added and the mixture was stirred for 2 h, then left standing at 30 °C for 20 h. The molar ratios in the mixture were 39:0.26:0.18:1 for H_2O:C_nTAB:TMAOH:SiO_2 respectively. Hydrothermal treatment was done by heating the mixture to 150, 170 or 180 °C in an autoclave for 44 h. The MCM-41 was subsequently washed and calcined as described for SBA-15. The silicas are referred to as MCM-41 followed by the C_n(TAB) chain length and the hydrothermal treatment temperature; the suffix '-2' is used to differentiate scaffolds prepared using the same procedure.

1.2 $LiBH_4/SiO_2$ nanocomposites

All silica scaffolds were dried at 300 °C in a glass reactor under a constant flow of inert gas, using a ramp of 5 °C/min, and kept at that temperature for 6 h. Subsequently, the dried silica was stored in gloveboxes with H_2O and O_2 levels typically $\leq$ 1 ppm.

The $LiBH_4/SiO_2$ nanocomposites were prepared via melt-infiltration of the dried silica scaffolds.[S6] $LiBH_4$ (95 %, Sigma-Aldrich) and dried silica were hand-mixed in the glovebox. The $LiBH_4$ loadings (fraction of pores that would be filled with $LiBH_4$ if all $LiBH_4$ infiltrates) can be found in tables S2 and S3 for the samples used for impedance spectroscopy and NMR experiments respectively. These mixtures were transferred to borosilicate glass vials and placed in a stainless-steel autoclave. Approximately 50 bar H_2 gas was applied and the autoclave was heated to 300 °C using a ramp of 3 °C/min, and kept at this temperature for 30 min. After cooling down, the H_2 pressure was released and the samples were transferred into the glovebox without exposure to air. Nanocomposites used for studies by NMR are named by their scaffold suffixed by ^{i} or ^{ii} to distinguish nanocomposites of different batches that use the same silica scaffold.

Table S1: Structural properties of the silica scaffolds.

Silica ID	S_{BET} [a]	V_{total} [b]	$V_{pore,meso}$ [c]	$V_{pore,micro}$ [c]	d_{BJH} [d]	d_{DFT} [e]	$t^{BJH}_{(100)}$ [f]	$t^{DFT}_{(100)}$ [f]	$t^{BJH}_{(200)}$ [f]	$t^{DFT}_{(200)}$ [f]
	m^2/g	mL/g	mL/g (%)	mL/g (%)	nm	nm	nm	nm	nm	nm
SBA-15-40	682	0.54	0.35 (65)	0.17 (31)	5.6	7.1	3.2	1.7	3.8	2.3
SBA-15-70	735	0.77	0.51 (67)	0.15 (19)	6.6	8.3	2.7	1.0	3.4	1.7
SBA-15-85	793	0.83	0.51 (62)	0.21 (25)	7.2	9.2	2.4	0.4	3.0	1.0
SBA-15-100	724	1.03	0.73 (71)	0.10 (10)	8.9	11.2	1.1	−1.2	1.7	−0.6
SBA-15-120	491	0.96	0.63 (66)	0.09 (9)	10.1	12.7	-	-	0.7	−1.9
MCM-41-C_{10}-150	845	0.69	0.44 (63)	0.00 (0)	1.9	3.0	2.3	1.2	2.5	1.4
MCM-41-C_{14}-150	979	0.86	0.78 (91)	0.00 (0)	2.7	4.0	1.9	0.6	1.9	0.6
MCM-41-C_{16}-140	1113	1.13	0.93 (83)	0.00 (0)	2.9	4.2	1.6	0.3	1.7	0.4
MCM-41-C_{16}-150	1083	1.04	0.96 (92)	0.00 (0)	3.1	4.5	1.7	0.3	1.8	0.4
MCM-41-C_{16}-165	550	0.55	0.40 (73)	0.07 (13)	3.9	5.3	2.1	0.7	3.2	1.8
MCM-41-C_{16}-170	474	0.46	0.33 (72)	0.06 (13)	3.7	5.1	2.3	0.9	2.5	1.1
MCM-41-C_{16}-180	346	0.36	0.33 (91)	0.00 (0)	3.3	4.6	2.9	1.6	3.3	2.0
MCM-41-C_{16}-150-2	1194	1.20	1.06 (88)	0.00 (0)	3.2	4.5	1.7	0.4	1.8	0.5
MCM-41-C_{16}-165-2	564	0.59	0.42 (72)	0.08 (13)	3.7	5.2	2.4	0.9	2.7	1.2
MCM-41-C_{16}-180-2	421	0.44	0.36 (83)	0.03 (6)	3.3	4.7	3.0	1.6	3.3	1.9
Aerosil 90	71	0.18	- (-)	0.02 (12)	-	-	-	-	-	-
Aerosil 300	278	0.89	- (-)	0.04 (5)	-	-	-	-	-	-
Aerosil 380	352	1.06	- (-)	0.05 (4)	-	-	-	-	-	-

[a] Specific surface area via the BET method.[S7] (N_2-physisorption)
[b] Total volume of pores at 0.997 P/P_0. (N_2-physisorption)
[c] Pore volumes determined using the t-plot method and the Harkins-Jura reference isotherm.[S8] (N_2-physisorption)
[d] Pore diameter via the classical BJH method on the adsorption isotherm.[S9] (N_2-physisorption)
[e] Pore diameter via density functional theory fit.[S10] (N_2-physisorption)
[f] The thickness t of the pore walls of MCM-41 and SBA-15 was obtained by relating the lattice spacing a_0 (XRD, (100) or (200) reflection) with the pore diameter d_{pore} (N_2-physisorption, DFT or BJH model) via $t = a_0 - d_{pore}$, where a_0 was obtained from XRD via $a_0 = 2d_{n00}/\sqrt{3}$ (n=1 or 2).[S1]. For SBA-15, the DFT model for cylindrical pores is less applicable, resulting in negative thickness values.

Table S2: Properties and ionic conductivity of the $LiBH_4/SiO_2$ nanocomposites used for conductivity measurements.

Silica ID	Loading (wt%)[a]	(%V_p)[b]	Confined (%)[c]	Conduct. (μS/cm)[d]	E_{act} (eV)[e]	Fig. 1 [f]	Pressure (MPa)[g]
SBA-15-40	32	134	82	7.1	0.51	✓	150
SBA-15-70	36	128	79	1.1	0.58	✓	150
SBA-15-85	38	128	73	6.5	0.58	✓	150
SBA-15-100	41	126	64	9.0	0.54	✓	150
SBA-15-120	37	123	76	5.6	0.52	✓	150
MCM-41-C_{14}-150	40	130	63	9.6	0.48	✓	150
MCM-41-C_{16}-140	44	129	50	17.1	0.48	✓	150
MCM-41-C_{16}-150	45	130	73	11.0	0.45	✓	150
MCM-41-C_{16}-150	52	169	64	17.6	0.46	✓	150
MCM-41-C_{16}-150-2	59	204	86	8.8	0.49		150
MCM-41-C_{16}-150-2	62	226	63	7.4	0.51		150
MCM-41-C_{16}-165	29	131	81	3.9	0.52		150
MCM-41-C_{16}-165-2	46	251	77	7.1	0.50		150
MCM-41-C_{16}-180	31	208	88	1.6	0.49		150
MCM-41-C_{16}-150-2	59	204	86	0.8	0.65		75
MCM-41-C_{16}-150-2	55	175	68	7.0	0.50		75
MCM-41-C_{16}-165-2	46	251	77	0.6	0.62		75
MCM-41-C_{16}-165-2	37	175	86	1.9	0.56		75
MCM-41-C_{16}-165-2	40	204	99	2.8	0.50		75
MCM-41-C_{16}-170	28	151	115	1.3	0.53		75
MCM-41-C_{16}-180-2	34	200	100	0.2	0.61		75
Aerosil 90	44	-	0 %[h]	1.0	0.58	✓	150
Aerosil 300	44	-	18 %[h]	4.5	0.57	✓	150
Aerosil 380	44	-	47 %[h]	2.0	0.55	✓	150

[a] Mass percentage of $LiBH_4$ in the nanocomposite.

[b] Theoretical fraction of silica pores filled with $LiBH_4$.

[c] Fraction of silica pores filled with $LiBH_4$ according to DSC.

[d] Ionic conductivity at 30 °C, interpolated from a linear fit of the Arrhenius plot between ambient temperature and 100 °C (figure S5).

[e] Apparent activation energy of the Li-ion hopping process, determined from a linear fit of the Arrhenius plot between ambient temperature and 100 °C (figure S5).

[f] A checkmark indicates whether the nanocomposite was included in Figure 1. Nanocomposites with excessive $LiBH_4$ loadings, thick walls (compression differences, see figure S17) or different pressures were excluded from this figure.

[g] The pressure for making the pellet consisting of lithium foil|nanocomposite|lithium foil, which was subsequently used in impedance spectroscopy.

[h] Fumed silica lacks a well-defined pore structure. Hence, the fraction of $LiBH_4$ that does no longer undergo the bulk phase transition around 110 °C is shown instead.

Table S3: Composition of the nanocomposites used in the studies with NMR. A suffix ^{i} or ^{ii} is added to the silica scaffold ID to distinguish two nanocomposites that use the same silica scaffold.

Nanocomposite<br>Silica ID + 'i'/'ii'	Loading<br>(wt%)[a]	 (%V_p)[b]	Confined<br>(%)[c]	Dynamic $LiBH_4$<br>(%)[d]
SBA-15-40^{i}	24	92	85	36
SBA-15-40^{ii}	24	90	86	27
SBA-15-85^{i}	30	91	75	19
SBA-15-85^{ii}	30	90	81	27
SBA-15-120^{i}	30	87	70	21
SBA-15-120^{ii}	30	87	70	17
MCM-41-C_{10}-150^{i}	21	90	37	19
MCM-41-C_{10}-150^{ii}	21	90	43	21
MCM-41-C_{14}-150^{i}	32	96	59	20
MCM-41-C_{14}-150^{ii}	32	95	64	19
MCM-41-C_{16}-150^{i}	37	90	68	13
MCM-41-C_{16}-150-2^{i}	39	90	59	13
MCM-41-C_{16}-170^{i}	19	91	82	33
MCM-41-C_{16}-180^{i}	17	93	91	53
MCM-41-C_{16}-180-2^{i}	19	91	79	43
Aerosil 90^{i}	11	-	40 %[e]	3
Aerosil 300^{i}	29	-	66 %[e]	4
Aerosil 300^{ii}	35	-	74 %[e]	3

[a] Mass percentage of $LiBH_4$ in the nanocomposite.
[b] Theoretical fraction of silica pores filled with $LiBH_4$.
[c] Fraction of silica pores filled with $LiBH_4$ according to DSC.
[d] Fraction of $LiBH_4$ in the most-dynamic fraction at 30 °C as determined via static ^{1}H NMR spectra (figure S6). The error is 4 percent point or less.
[e] Fumed silica lacks a well-defined pore structure. Hence, the fraction of $LiBH_4$ that does no longer undergo the bulk phase transition around 110 °C is shown instead.

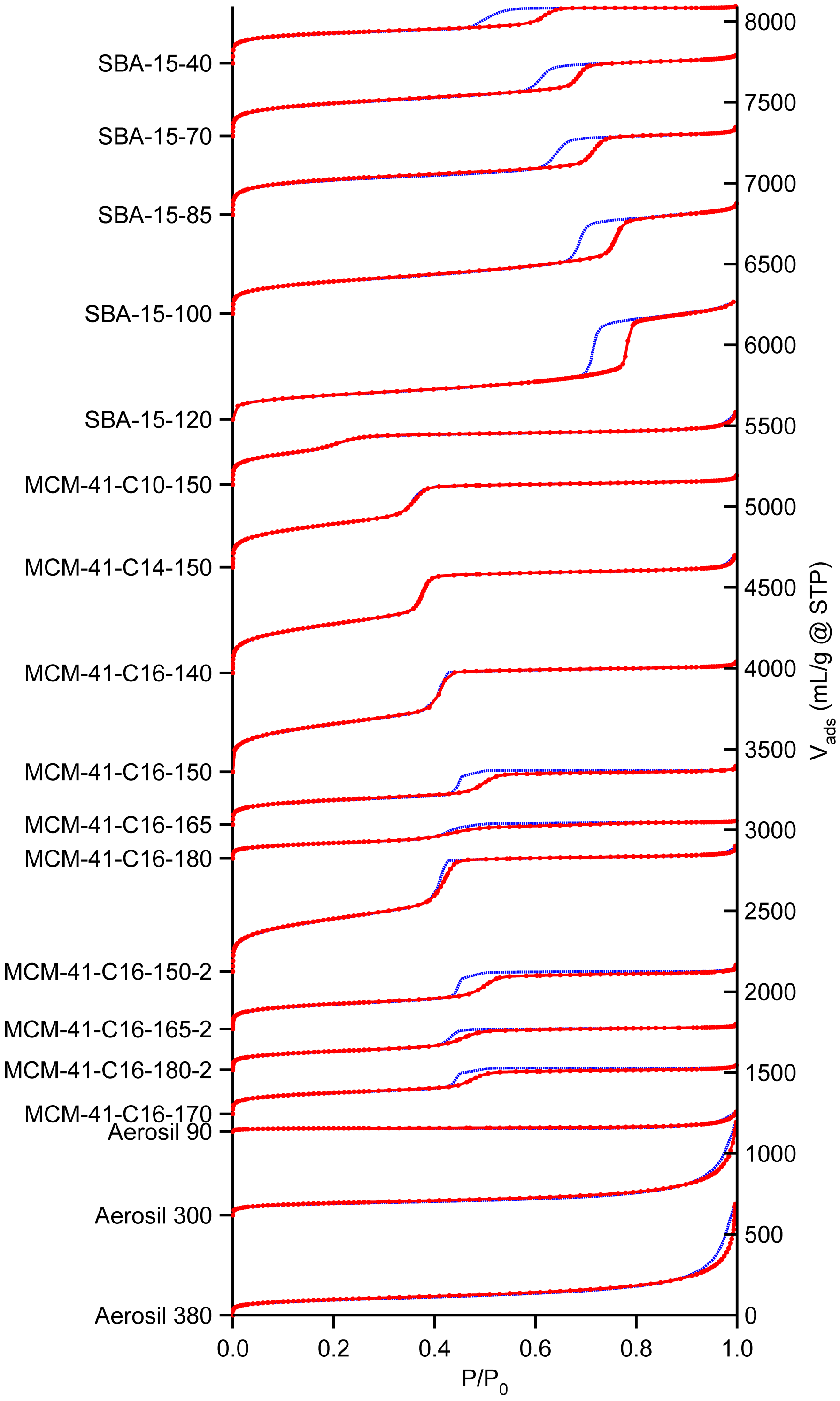

Figure S1: N_2-physisorption isotherms of the silicas. Adsorption isotherms are shown in red, desorption isotherms in blue. An arbitrary offset was applied for clarity.

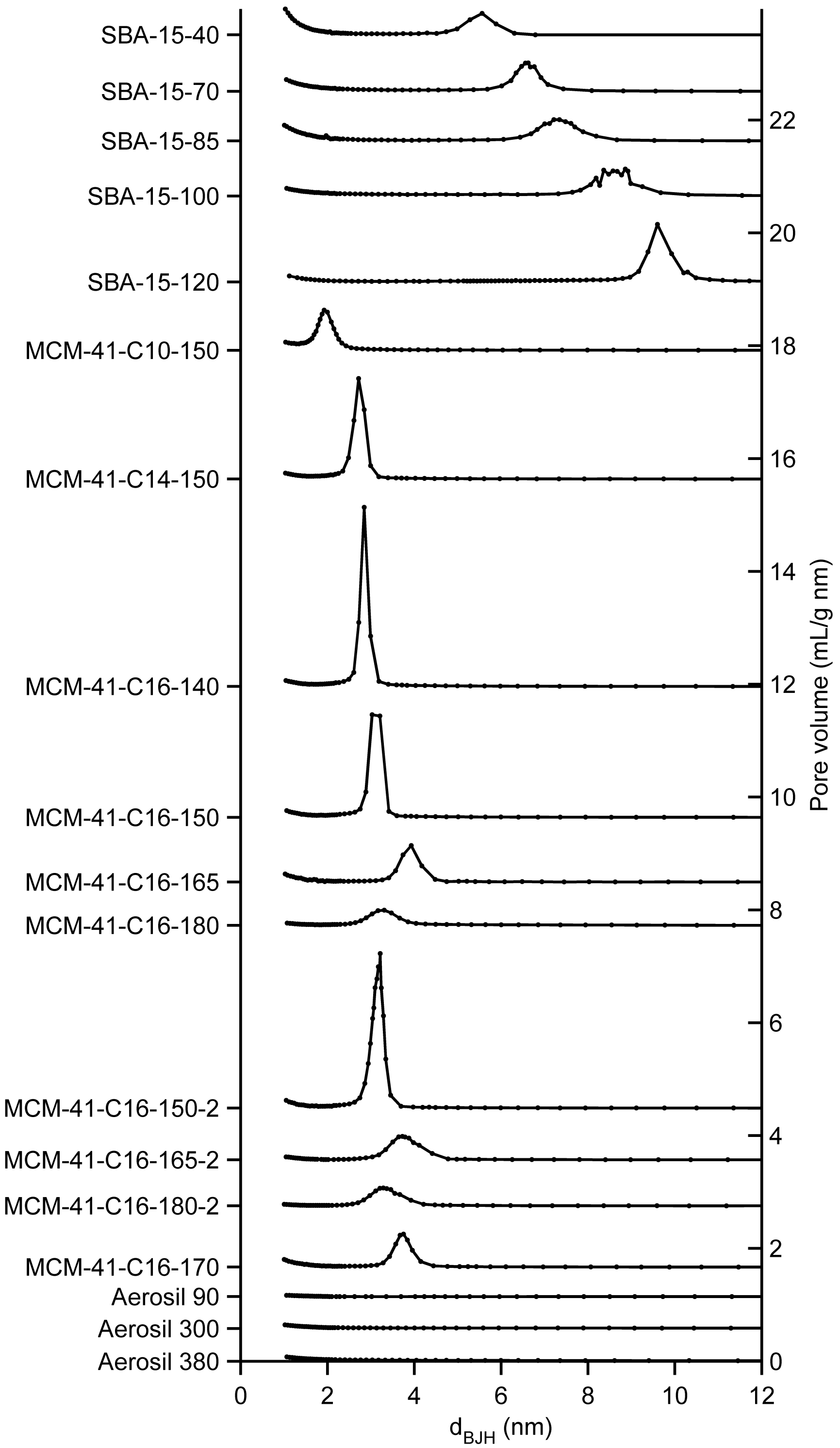

Figure S2: Pore size distributions of the silicas according to the classical BJH model[S9] applied to the adsorption isotherm (figure S1).

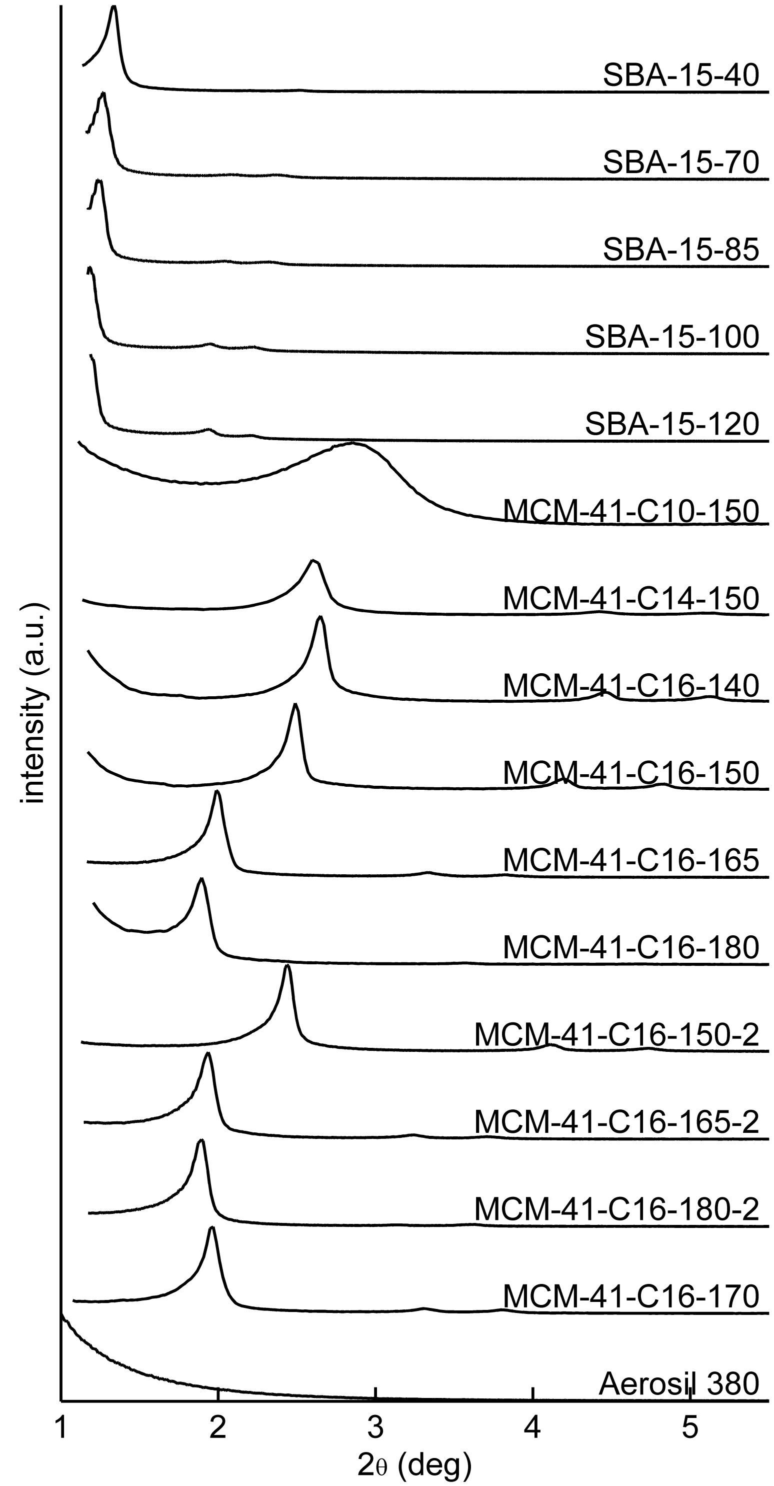

Figure S3: Normalized low angle powder X-ray diffraction patterns of the silica scaffolds. The major peaks of MCM-41 and SBA-15 correspond, from left to right, to the (100), (110) and (200) reflections, respectively. Higher reflections are visible for some silica scaffolds. Aerosil has no long-range ordering and is hence featureless. An arbitrary offset was applied for clarity. The sloping baseline on the left is an artifact due to direct beam exposure.

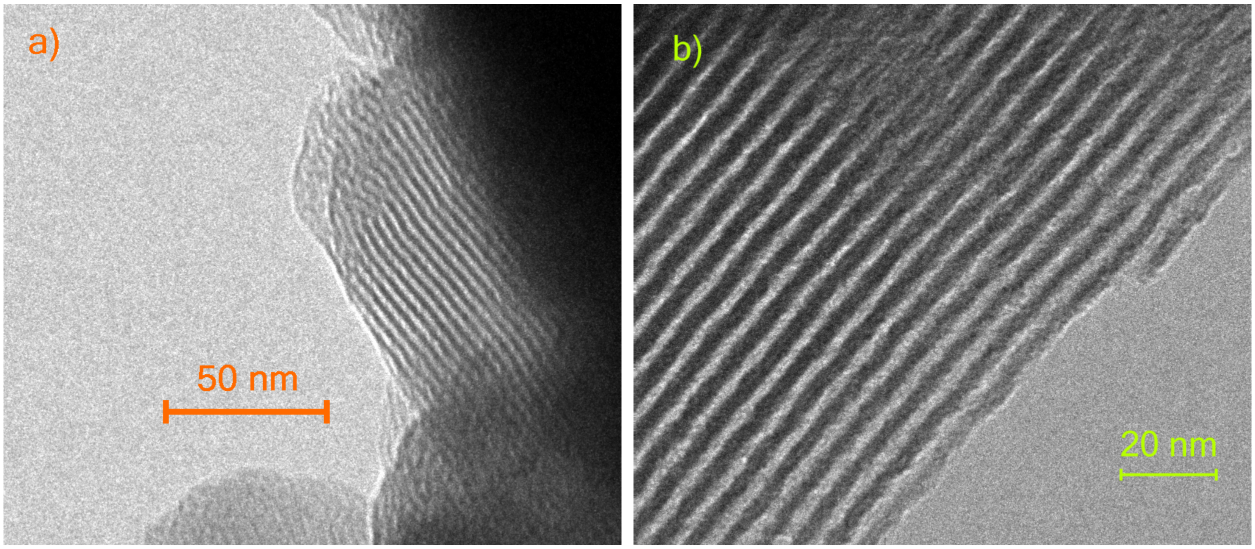

Figure S4: Transmission electron micrograms of (a) MCM-41-C_{16}-150 and (b) MCM-41-C_{16}-180, showing the ordered porous structure of both silica scaffolds. Mesoporous silica scaffolds are very prone to electron beam damage[S11], hence different maximum zoom levels were used. Some beam damage is visible in b).

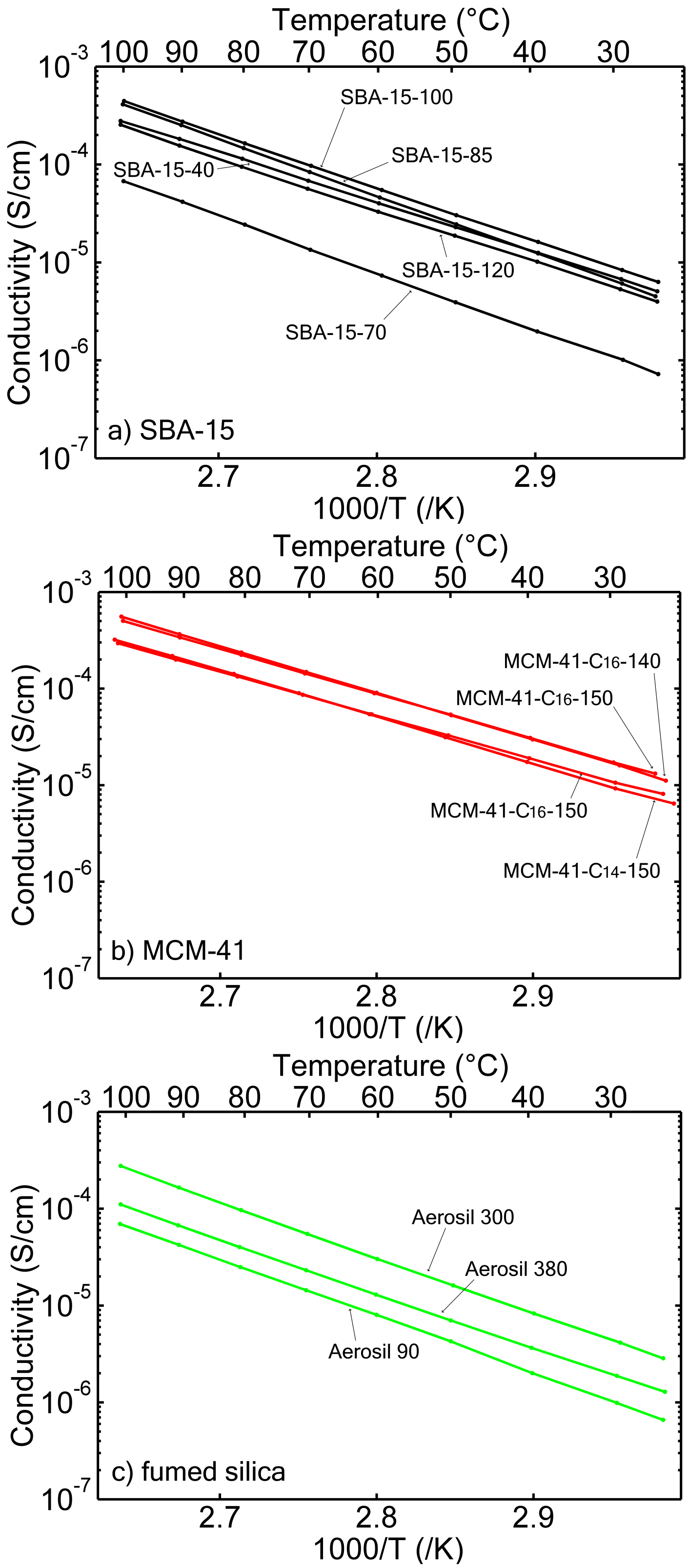

Figure S5: Arrhenius plots of the ionic conductivities of nanocomposites using (a) SBA-15, (b) MCM-41 and (c) fumed silica (Aerosil) scaffolds as function of temperature, as shown in figure 1 and listed in table S2.

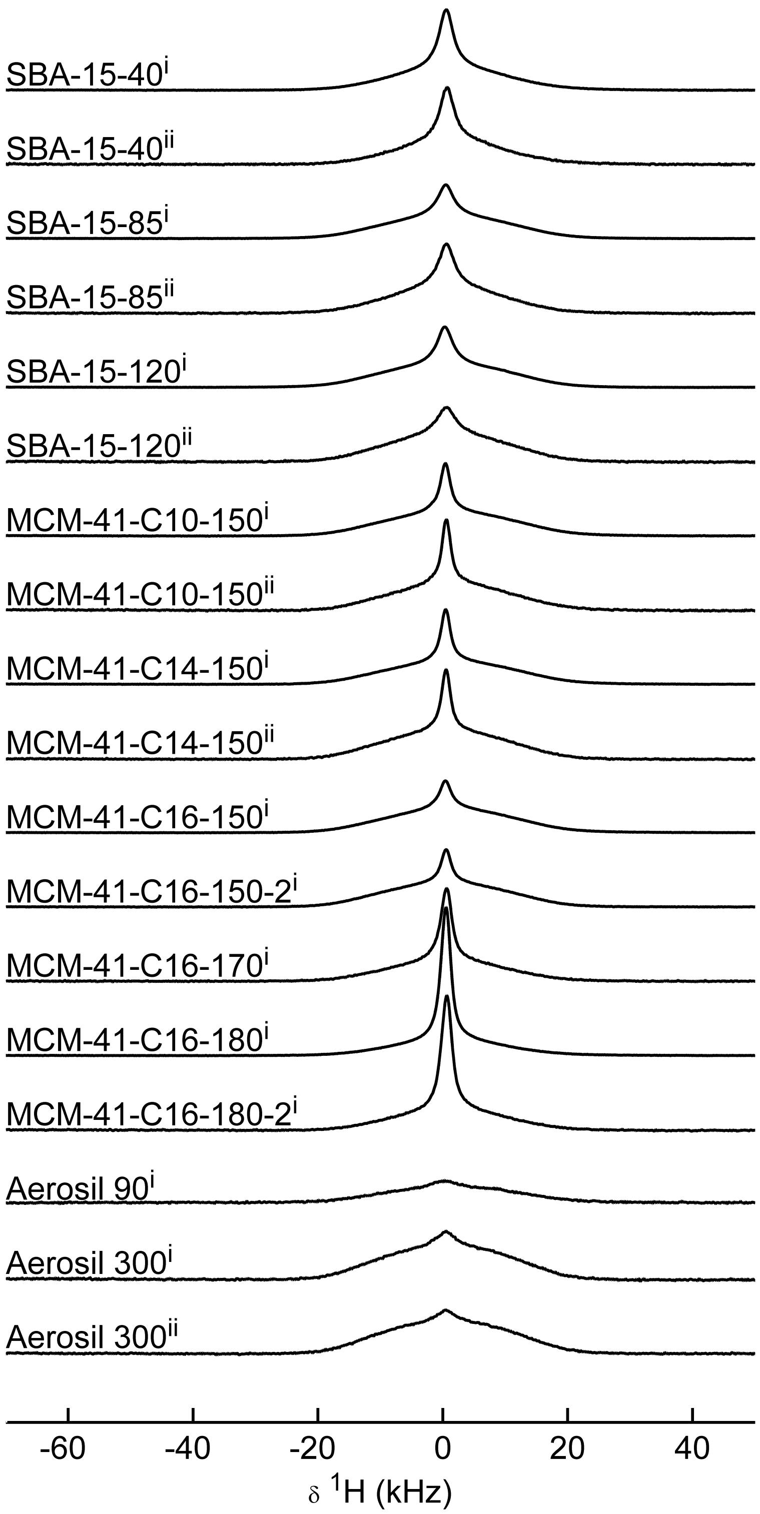

Figure S6: Normalized ^{1}H NMR spectra of nanocomposites, measured at a field of 7.05 T.

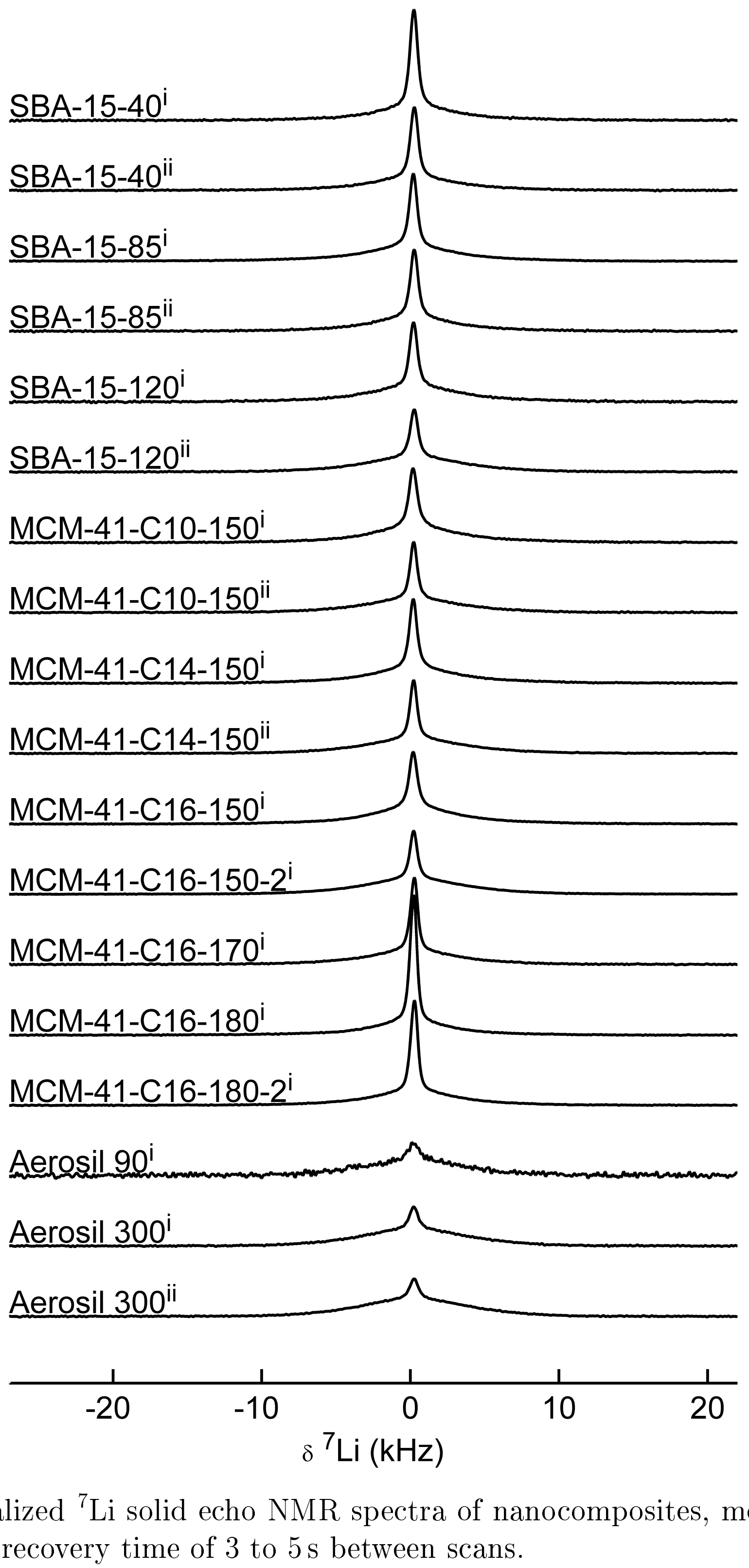

Figure S7: Normalized ^{7}Li solid echo NMR spectra of nanocomposites, measured at a field of 7.05 T, using a recovery time of 3 to 5 s between scans.

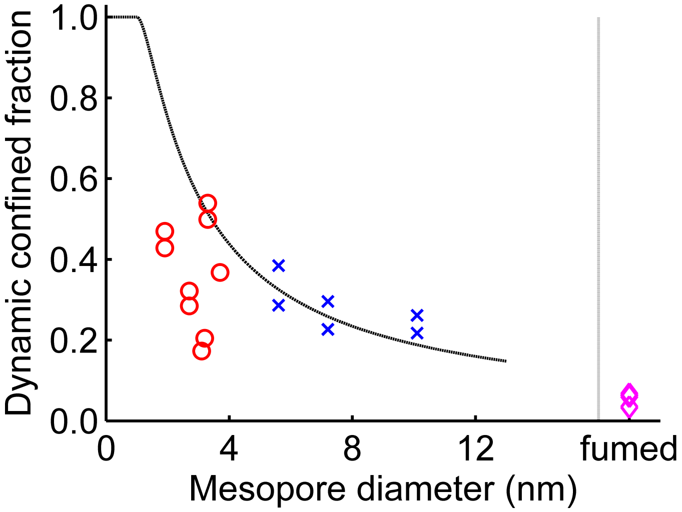

Figure S8: Plot of the fraction of mobile ions of the nanocomposites at 30 °C as function of the mesopore diameter of their silica scaffolds. MCM-41-based nanocomposites are denoted by circles, SBA-15-based by crosses and those based upon fumed silica (which has no well-defined mesopores) by diamonds. The black line indicates the hypothetical fraction of mobile ions for perfectly cylindrical pores as function of the pore diameter d via $4\,(td - t^2)\,/d^2$, assuming a mobile layer thickness t of 0.5 nm (as found in our previous study for SBA-15-based nanocomposites[S12]). Clearly this hypothetical model does not cover the data points of nanocomposites based on MCM-41.

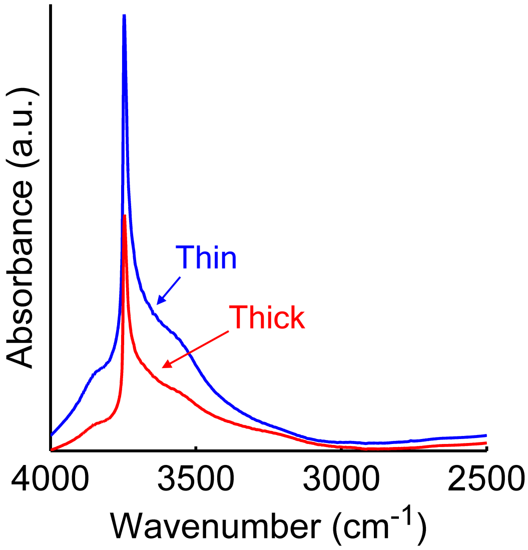

Figure S9: Diffuse reflectance infrared (DRIFTS) spectra of thin-walled (MCM-41-C_{16}-150-2) and thick-walled (MCM-41-C_{16}-180-2) silica after being dried at 300 °C. The peaks are assigned to isolated (about 3745 cm^{-1}) and vicinal (hydrogen-bonded, 3700-3000 cm^{-1}) silanol O – H vibrations, in accordance with literature.[S13] There are no peaks in the C – H stretching region (3000-2900 cm^{-1}), indicating a proper removal of the organic template during calcination.

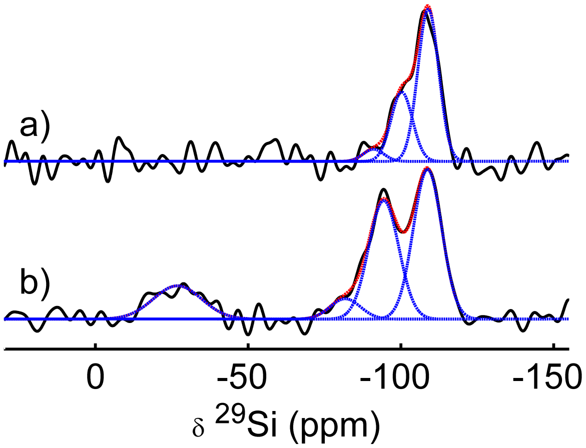

Figure S10: ^{29}Si MAS NMR spectra of (a) the silica scaffold MCM-41-C$_{16}$-150-2 and (b) the nanocomposite MCM-41-C$_{16}$-150-2^{i}, as well as their deconvolutions into individual peaks. For assignment of the peaks, the reader is referred to figure 3 and the main text; the ratios of the peaks are listed in table 2. The experiments were performed on a field of 7.05 T at 3 and 6.5 kHz MAS respectively. The spectrum of the silica (a) is the sum of spectra with a fixed delay of 50000 and 86400 s between subsequent scans. The ^{29}Si spin-relaxation time, which is longest for the Q_4 peak, is $T_1^{Q_4} \approx 2 \cdot 10^4 \pm 1 \cdot 10^4$ s in this silica scaffold. The spectrum of the nanocomposite (b) was recorded with a fixed delay of 3600 s between scans; at longer delays between scans, (only) the Q_4 peak will gain more intensity (4 to 8 percent points), presumably due to silica that is at a large distance from $LiBH_4$.

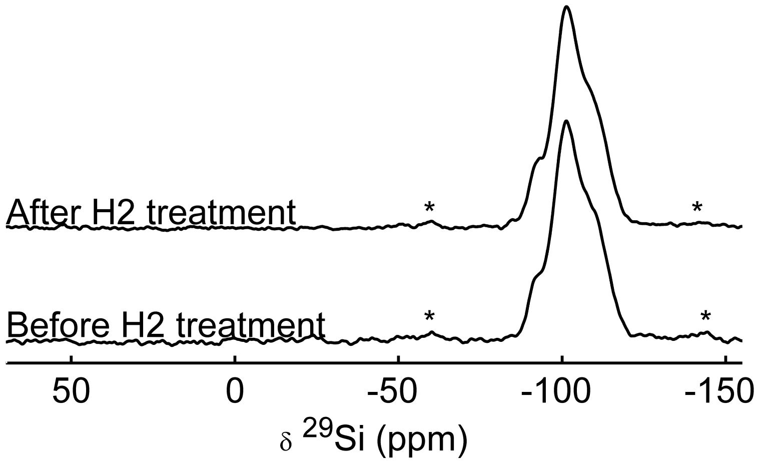

Figure S11: $\{^1H\}^{29}$Si CP-MAS NMR spectra of silica of the type SBA-15, before and after being treated under melt-infiltration conditions (without $LiBH_4$). The spectra display excellent agreement, indicating no changes have occurred as a result of this treatment. The spectra were recorded on a field of 9.4 T at a spinning speed of 3.25 kHz. The weak spinning sidebands are indicated by asterisks.

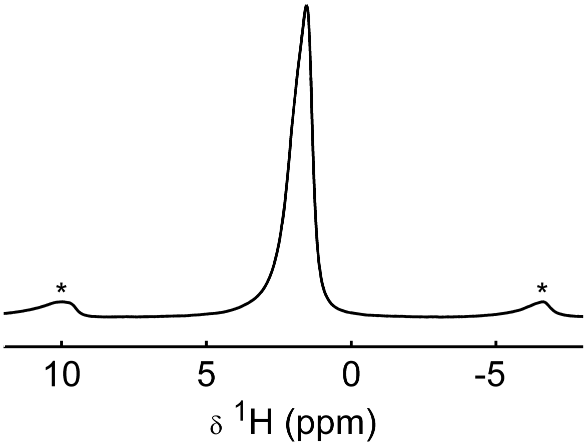

Figure S12: ^{1}H NMR spectrum of silica scaffold MCM-41-C_{16}-150-2 (without $LiBH_4$), measured using a Hahn echo at a field of 9.4 T under 3.25 kHz MAS. Spinning sidebands are indicated by asterisks. The peaks between 1.6 and 2.2 ppm are ascribed to silanol groups.[S14]

Table S4: Dipolar second moments and corresponding (r^{-3}-weighted) average internuclear distances obtained from fits of $\{^{7}Li\}^{29}Si$ and $\{^{11}B\}^{29}Si$ REDOR curves of the nanocomposite MCM-41-C_{16}-150-2^{i} (figure S13). The REDOR curves were fitted to the analytical formula derived by Hirschinger to obtain the heteronuclear dipolar second moment and the average internuclear distance.[S15–S17] As the quadrupolar interactions of ^{11}B and ^{7}Li in $LiBH_4$/SiO_2 nanocomposites are small, the REDOR experiment can be used quantitatively.[S18] The margin in the least-squares fit of the second moment is typically 20-30 %, resulting in an error of approximately 0.3 Å in the distance. This excludes the error in the REDOR curve, and assumes each lithium or boron atom couples with only one silicon atom of a certain species. The data of $Q_3^{'}$ may include a contribution of isolated SiOH or SiH sites, but this contribution is expected to be small.

Nuclei	Second moment (kHz^2)			Internuclear distance (Å)		
	mii	$Q_3^{'}$	Q_4	*mii*	$Q_3^{'}$	Q_4
^{29}Si–^{7}Li	2.1	2.3	0.25	3.4	3.4	4.9
^{29}Si–^{11}B	1.1	0.23	0.21	3.6	4.6	4.7

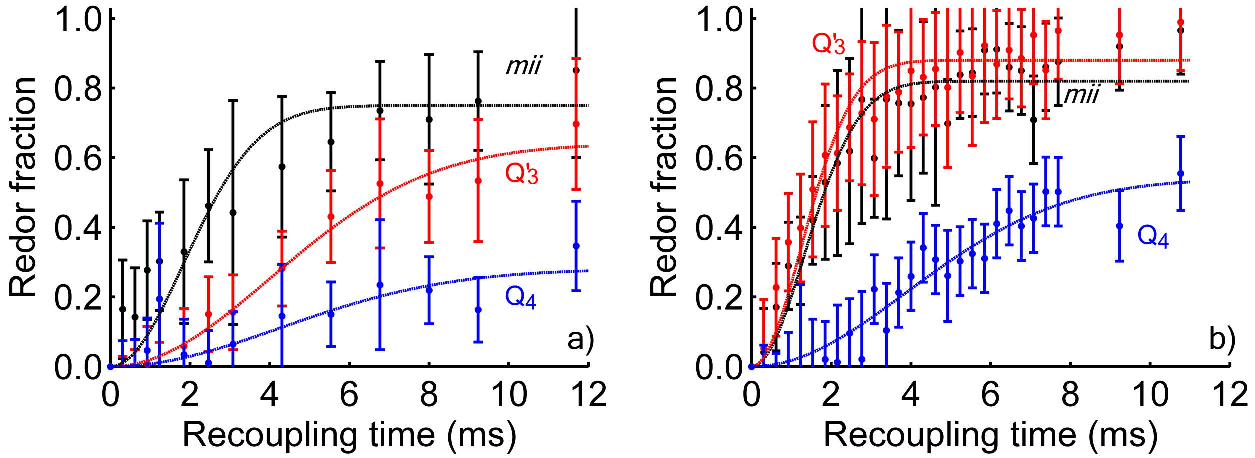

Figure S13: Least-squares fits of the (a) $\{^{11}B\}^{29}Si$ and (b) $\{^{7}Li\}^{29}Si$ REDOR difference curves of $LiBH_4$/SiO_2 nanocomposites, shown in figure 5. The curves were fitted to the formula derived by Hirschinger[S15], and assume a single, homogeneous dipolar interaction with either ^{11}B or ^{7}Li per silicon site. Although the error bars are significant, visual inspection suggests the fits underestimate the curve at longer recoupling times for *mii* and $Q_3^{'}$ sites, which is likely the result of inhomogeneity in the dipolar couplings.

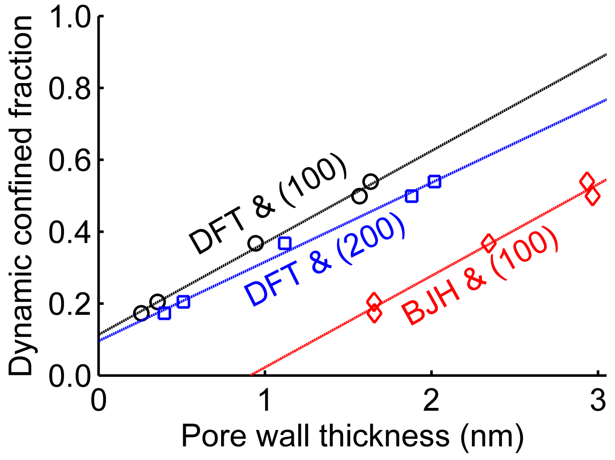

Figure S14: Figure showing the effect of the physisorption pore size distribution model (DFT or BJH) and the influence of the XRD reflection (either (100) or (200)) used to calculate the pore wall thickness. The black circles are equal to the data shown in figure 6.

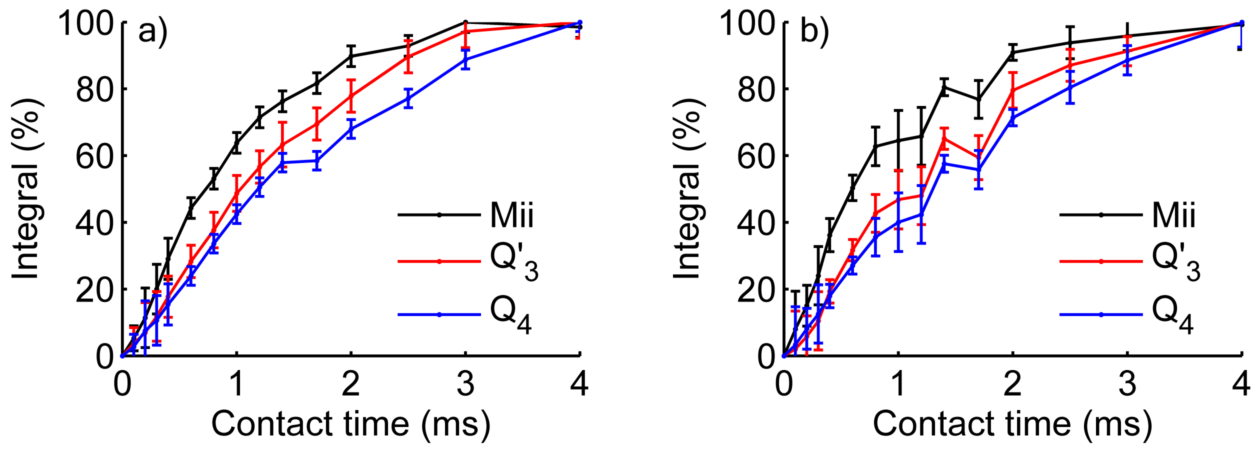

Figure S15: $\{^1H\}^{29}Si$ LGCP build-up curves of (a) thin (MCM-41-C_{16}-150-2^i) and (b) thick (MCM-41-C_{16}-180^i) walled MCM-41-based nanocomposites, normalized to the maximal peak integral, or 4 ms if the maximum was not reached yet. The spectra were measured at a field of 9.4 T under 6.5 kHz MAS.

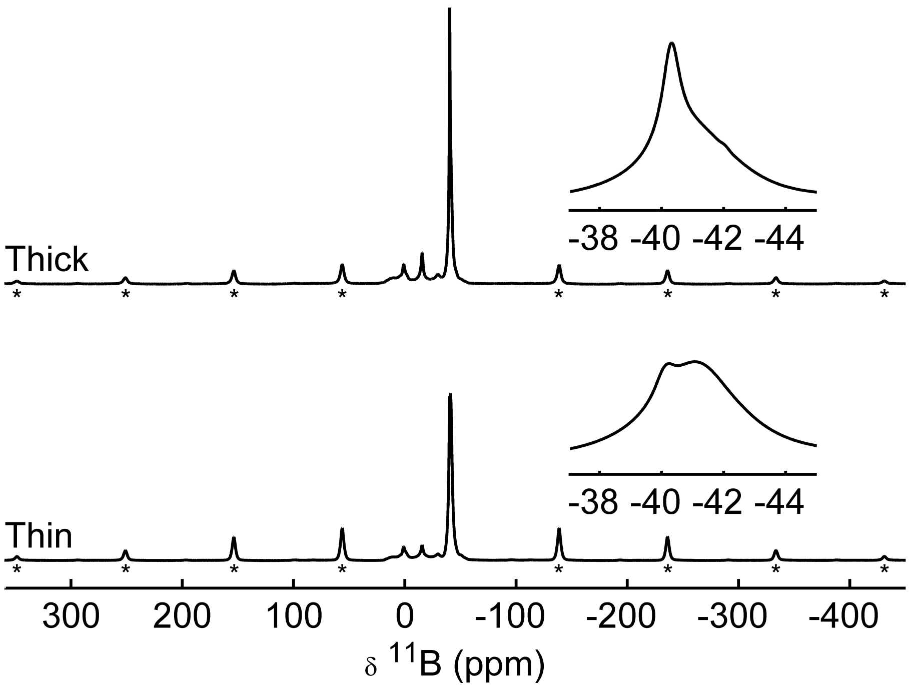

Figure S16: Normalized ^{11}B MAS NMR spectra of thin (MCM-41-C_{16}-150-2^{i}) and thick (MCM-41-C_{16}-180-2^{i}) walled MCM-41-based nanocomposites. The experiment was performed at 12.5 kHz MAS in a field of 9.4 T. Insets contain the spectrum in the region of the BH_4^- peaks. Spinning sidebands of $LiBH_4$ are indicated by asterisks. The small peaks between 20 and −35 ppm correspond mostly to oxidations of $LiBH_4$ as demonstrated in the supporting information of our previous study.[S12] The highly dynamic and more bulk-like $LiBH_4$ fractions resonate at −40.5 and −41.3 ppm, respectively.[S19] The chemical shift of the bulk-like fraction is equal to the chemical shift of bulk $LiBH_4$.[S20]

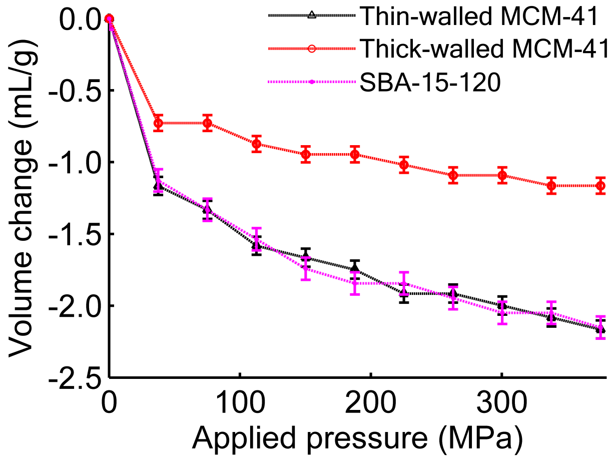

Figure S17: Change in volume of the silica upon applying pressure. The volume change was measured in-situ in a 13 mm pellet die. The silicas shown are MCM-41-C_{16}-150-2 (thin-walled MCM-41), MCM-41-C_{16}-180-2 (thick-walled MCM-41) and SBA-15-120. For comparison: the volume change of Aerosil 380 (not shown) was about −8 mL/g at a pressure of 38 MPa, then stabilizing at −8.5 mL/g at higher pressures.

Semi-quantitative approach to determine whether the *mii*-peak is proportional to the interface between $LiBH_4$ and silica or to the total composition of the nanocomposite.

Although CP-MAS experiments are not inherently quantitative, it is possible to do a rough quantitative comparison of the *mii*-peaks in the nanocomposites MCM-41-C_{16}-150-2^i (thin pore walls) and MCM-41-C_{16}-180-2^i (thick pore walls), as the cross-polarization dynamics are comparable for both nanocomposites (figure S15).

After normalization to equal number of molecules per NMR rotor (using the ^7Li integrals and the sample composition) and the number of scans, the ratio between the *mii*-peaks of the nanocomposites with thin and thick silica pore walls was:

$$\frac{I^{mii}_{thin}}{I^{mii}_{thick}} \approx 0.5$$

We now consider two hypothetical cases:

1. **The *mii*-peak is proportional to the surface area of the silica**. In this case, the peak would be expected to scale with the surface area (BET, m^2/mole) of the silica scaffold, and the molar ratio between silica and $LiBH_4$ ($f^{SiO2} = n_{SiO2} \div (n_{LiBH4} + n_{SiO2}) \propto n_{SiO2}$, the latter step due to normalization), i.e., doubling of the surface area per amount of silica or doubling the amount of silica (which would both double the total surface area) would result in a doubling of the *mii* peak. This assumes sufficient $LiBH_4$ is present to wet the entire silica surface. This hypothetical case would yield a ratio of:
$$\frac{I^{mii}_{thin}}{I^{mii}_{thick}} \propto \frac{BET_{thin} \cdot f^{SiO2}_{thin}}{BET_{thick} \cdot f^{SiO2}_{thick}} \approx 1.9$$

2. **The *mii*-peak is proportional to the total amount of silica**. In this case, the peak would roughly scale with the fraction of silica in the nanocomposite. This case would yield a hypothetical ratio of:
$$\frac{I^{mii}_{thin}}{I^{mii}_{thick}} \propto \frac{f^{SiO2}_{thin}}{f^{SiO2}_{thick}} \approx 0.7$$

Clearly, the hypothetical ratio for an interaction involving the total amount of silica is much closer to the experimentally determined ratio, than an interaction involving only the interface between lithium borohydride and silica. It should be noted that these are rough approximations: i.e. pore filling deficiencies are not taken into account.

References

(S1) Zhao, D.; Feng, J.; Huo, Q.; Melosh, N.; Fredrickson, G. H.; Chmelka, B. F.; Stucky, G. D. Triblock Copolymer Syntheses of Mesoporous Silica with Periodic 50 to 300 Angstrom Pores. *Science* **1998**, *279*, 548–552.

(S2) Galarneau, A.; Cambon, H.; Di Renzo, F.; Fajula, F. True Microporosity and Surface Area of Mesoporous SBA-15 Silicas as a Function of Synthesis Temperature. *Langmuir* **2001**, *17*, 8328–8335.

(S3) Beck, J. S.; Vartuli, J. C.; Roth, W. J.; Leonowicz, M. E.; Kresge, C. T.; Schmitt, K. D.; Chu, C. T.-W.; Olson, D. H.; Sheppard, E. W.; McCullen, S. B.; Higgins, J. B.; Schlenker, J. L. A New Family of Mesoporous Molecular Sieves Prepared with Liquid Crystal Templates. *J. Am. Chem. Soc.* **1992**, *114*, 10834–10843.

(S4) Cheng, C.-F.; Zhou, W.; Ho Park, D.; Klinowski, J.; Hargreaves, M.; Gladden, L. F. Controlling the Channel Diameter of the Mesoporous Molecular Sieve MCM-41. *J. Chem. Soc., Faraday Trans.* **1997**, *93*, 359–363.

(S5) Sangchoom, W.; Mokaya, R. High Temperature Synthesis of Exceptionally Stable Pure Silica MCM-41 and Stabilisation of Calcined Mesoporous Silicas via Refluxing in Water. *J. Mater. Chem.* **2012**, *22*, 18872.

(S6) Ngene, P.; Adelhelm, P.; Beale, A. M.; de Jong, K. P.; de Jongh, P. E. $LiBH_4$/SBA-15 Nanocomposites Prepared by Melt Infiltration under Hydrogen Pressure: Synthesis and Hydrogen Sorption Properties. *J. Phys. Chem. C* **2010**, *114*, 6163–6168.

(S7) Brunauer, S.; Emmett, P. H.; Teller, E. Adsorption of Gases in Multimolecular Layers. *J. Am. Chem. Soc.* **1938**, *60*, 309–319.

(S8) Jura, G.; Harkins, W. D. A New Adsorption Isotherm Which Is Valid Over a Very Wide Range of Pressure. *J. Chem. Phys.* **1943**, *11*, 430–431.

(S9) Barrett, E. P.; Joyner, L. G.; Halenda, P. P. The Determination of Pore Volume and Area Distributions in Porous Substances. I. Computations from Nitrogen Isotherms. *J. Am. Chem. Soc.* **1951**, *73*, 373–380.

(S10) Jaroniec, M.; Kruk, M.; Olivier, J. P.; Koch, S. In *Characterisation of Porous Solids V*; Unger, K. K., Kreysa, G., Baselt, J., Eds.; Stud. Surf. Sci. Catal.; Elsevier, 2000; Vol. 128; pp 71–80.

(S11) Blanford, C. F.; Carter, C. B. Electron Radiation Damage of MCM-41 and Related Materials. *Microsc. Microanal.* **2003**, *9*, 245–263.

(S12) Lambregts, S. F. H.; van Eck, E. R. H.; Suwarno,; Ngene, P.; de Jongh, P. E.; Kentgens, A. P. M. Phase Behavior and Ion Dynamics of Nanoconfined $LiBH_4$ in Silica. *J. Phys. Chem. C* **2019**, *123*, 25559–25569.

(S13) Gallas, J.-P.; Goupil, J.-M.; Vimont, A.; Lavalley, J.-C.; Gil, B.; Gilson, J.-P.; Miserque, O. Quantification of Water and Silanol Species on Various Silicas by Coupling IR Spectroscopy and in-Situ Thermogravimetry. *Langmuir* **2009**, *25*, 5825–5834.

(S14) Grünberg, B.; Emmler, T.; Gedat, E.; Shenderovich, I.; Findenegg, G. H.; Limbach, H.-H.; Buntkowsky, G. Hydrogen Bonding of Water Confined in Mesoporous Silica MCM-41 and SBA-15 Studied by ^{1}H Solid-State NMR. *Chem. - Eur. J.* **2004**, *10*, 5689–5696.

(S15) Hirschinger, J. Analytical Solutions to Several Magic-Angle Spinning NMR Experiments. *Solid State Nucl. Magn. Reson.* **2008**, *34*, 210–223.

(S16) Van Vleck, J. H. The Dipolar Broadening of Magnetic Resonance Lines in Crystals. *Phys. Rev.* **1948**, *74*, 1168–1183.

(S17) Abragam, A. *The Principles of Nuclear Magnetism*, 1st ed.; The International Series of Monographs on Physics; Oxford University Press: New York, 1961; Chapter 4.

(S18) Gullion, T.; Vega, A. J. Measuring Heteronuclear Dipolar Couplings for I=1/2, S>1/2 Spin Pairs by REDOR and REAPDOR NMR. *Prog. Nucl. Magn. Reson. Spectrosc.* **2005**, *47*, 123–136.

(S19) Verkuijlen, M. H. W.; Ngene, P.; de Kort, D. W.; Barré, C.; Nale, A.; van Eck, E. R. H.; van Bentum, P. J. M.; de Jongh, P. E.; Kentgens, A. P. M. Nanoconfined $LiBH_4$ and Enhanced Mobility of Li^+ and BH_4^- Studied by Solid-State NMR. *J. Phys. Chem. C* **2012**, *116*, 22169–22178.

(S20) Arnbjerg, L. M.; Ravnsbæk, D. B.; Filinchuk, Y.; Vang, R. T.; Cerenius, Y.; Besenbacher, F.; Jørgensen, J.-E.; Jakobsen, H. J.; Jensen, T. R. Structure and Dynamics for $LiBH_4$-LiCl Solid Solutions. *Chem. Mater.* **2009**, *21*, 5772–5782.
